# First mitochondrial genome-wide association study with metabolomics

**DOI:** 10.1093/hmg/ddab312

**Published:** 2021-10-27

**Authors:** Brahim Aboulmaouahib, Gabi Kastenmüller, Karsten Suhre, Sebastian Zöllner, Hansi Weissensteiner, Cornelia Prehn, Jerzy Adamski, Christian Gieger, Rui Wang-Sattler, Peter Lichtner, Konstantin Strauch, Antònia Flaquer

**Affiliations:** Institute for Medical Information Processing, Biometry and Epidemiology—IBE, LMU, Munich, Germany; Institute of Medical Biostatistics, Epidemiology and Informatics (IMBEI), University Medical Center, Johannes Gutenberg University, Mainz, Germany; Institute of Computational Biology, Helmholtz Zentrum München—German Research Center for Environmental Health, Neuherberg, Germany; Department of Physiology and Biophysics, Weill Cornell Medical College in Qatar, Qatar, State of Qatar; Department of Computational Medicine and Bioinformatics, University of Michigan, Ann Arbor, MI, USA; Department of Psychiatry, University of Michigan, Ann Arbor, MI, USA; Department of Genetics and Pharmacology, Institute of Genetic Epidemiology, Medical University of Innsbruck, Innsbruck, Austria; Metabolomics and Proteomics Core, Helmholtz Zentrum München–German Research Center for Environmental Health, Neuherberg, Germany; Institute of Experimental Genetics, Helmholtz Zentrum München–German Research Center for Environmental Health, Neuherberg, Germany; Department of Biochemistry, Yong Loo Lin School of Medicine, National University of Singapore, 8 Medical Drive, Singapore 117597, Singapore; Institute of Biochemistry, Faculty of Medicine, University of Ljubljana, Vrazov trg 2, 1000 Ljubljana, Slovenia; Research Unit of Molecular Epidemiology, Helmholtz Zentrum München—German Research Center for Environmental Health, Neuherberg, Germany; German Center for Diabetes Research (DZD), Neuherberg, Germany; Institute of Epidemiology, Helmholtz Zentrum München—German Research Center for Environmental Health, Neuherberg, Germany; Research Unit of Molecular Epidemiology, Helmholtz Zentrum München—German Research Center for Environmental Health, Neuherberg, Germany; German Center for Diabetes Research (DZD), Neuherberg, Germany; Institute of Epidemiology, Helmholtz Zentrum München—German Research Center for Environmental Health, Neuherberg, Germany; Institute of Human Genetics, Helmholtz Zentrum München—German Research Center for Environmental Health, Neuherberg, Germany; Institute for Medical Information Processing, Biometry and Epidemiology—IBE, LMU, Munich, Germany; Institute of Medical Biostatistics, Epidemiology and Informatics (IMBEI), University Medical Center, Johannes Gutenberg University, Mainz, Germany; Institute of Genetic Epidemiology, Helmholtz Zentrum München—German Research Center for Environmental Health, Neuherberg, Germany; Institute for Medical Information Processing, Biometry and Epidemiology—IBE, LMU, Munich, Germany; Institute of Genetic Epidemiology, Helmholtz Zentrum München—German Research Center for Environmental Health, Neuherberg, Germany

## Abstract

In the era of personalized medicine with more and more patient-specific targeted therapies being used, we need reliable, dynamic, faster and sensitive biomarkers both to track the causes of disease and to develop and evolve therapies during the course of treatment. Metabolomics recently has shown substantial evidence to support its emerging role in disease diagnosis and prognosis. Aside from biomarkers and development of therapies, it is also an important goal to understand the involvement of mitochondrial DNA (mtDNA) in metabolic regulation, aging and disease development. Somatic mutations of the mitochondrial genome are also heavily implicated in age-related disease and aging. The general hypothesis is that an alteration in the concentration of metabolite profiles (possibly conveyed by lifestyle and environmental factors) influences the increase of mutation rate in the mtDNA and thereby contributes to a range of pathophysiological alterations observed in complex diseases. We performed an inverted mitochondrial genome-wide association analysis between mitochondrial nucleotide variants (mtSNVs) and concentration of metabolites. We used 151 metabolites and the whole sequenced mitochondrial genome from 2718 individuals to identify the genetic variants associated with metabolite profiles. Because of the high coverage, next-generation sequencing-based analysis of the mitochondrial genome allows for an accurate detection of mitochondrial heteroplasmy and for the identification of variants associated with the metabolome. The strongest association was found for mt715_G > A_ located in the MT-12SrRNA with the metabolite ratio of C2/C10:1 (*P*-value = 6.82^*^10^−09^, *β* = 0.909). The second most significant mtSNV was found for mt3714_A > G_ located in the MT-ND1 with the metabolite ratio of phosphatidylcholine (PC) ae C42:5/PC ae C44:5 (*P*-value = 1.02^*^10^−08^, *β* = 3.631). A large number of significant metabolite ratios were observed involving PC aa C36:6 and the variant mt10689_G > A_, located in the MT-ND4L gene. These results show an important interconnection between mitochondria and metabolite concentrations. Considering that some of the significant metabolites found in this study have been previously related to complex diseases, such as neurological disorders and metabolic conditions, these associations found here might play a crucial role for further investigations of such complex diseases. Understanding the mechanisms that control human health and disease, in particular, the role of genetic predispositions and their interaction with environmental factors is a prerequisite for the development of safe and efficient therapies for complex disorders.

## Introduction

Genome-wide association studies (GWASs) have identified a number of genetic polymorphisms that convey an increased risk for developing diseases (1). However, the results of GWAS have not fully responded to initial expectations. The biological effect of many genes found associated with a particular disease cannot be explained so far. More surprising is the fact that, even when combining all available GWAS on a particular disorder, polymorphisms usually associated explain <5–10% of the risk of disease (1). However, by only associating genotypes with clinical outcomes, little can be inferred about the underlying disease-causing mechanisms. On the other hand, GWAS with metabolic traits as intermediate phenotypes (GWAS-metabolomics) have identified genetically determined variants in metabolic phenotypes that exhibit large effect sizes (2,3).

Recently, GWAS on the mitochondrial genome (mtGWAS) have also provided some new insights into the mechanisms underlying several complex diseases (4–6). Genetic variants that associate with changes in the homeostasis of key lipids, carbohydrates or amino acids are not only expected to display much larger effect sizes owing to their direct involvement in metabolite conversion and modification but may also provide more details on potentially affected pathways and may be more directly related to the etiology of the disease (2).

Mitochondrial DNA (mtDNA), which has a length of ~16.6 kb, codes for 13 subunits of the mitochondrial respiratory chain complexes, 2 ribosomal (rRNA) genes and 22 transfer RNA (tRNA) genes that are required for mitochondrial protein synthesis. Mitochondria consume oxygen and substrates to generate energy in the form of adenosine triphosphate (ATP) while producing reactive oxygen species (ROS), also known as free radicals, in the process. While ROS have important roles in cell signaling and homeostasis, ROS levels can increase drastically and cause significant damage to the DNA, thereby altering the membrane permeability and calcium homeostasis along with increasing the heteroplasmic mtDNA. This damage is termed oxidative stress. High levels of oxidative stress may damage cellular structures which, in turn, can lead to mitochondrial dysfunction, possibly even to apoptosis.

Mitochondrial mutations can either be somatic or inherited through the maternal line (7). Since many mtDNA copies are present in a cell and as owing to their high mutation rate [on average, 20 times higher than for nuclear DNA (nDNA)], new somatic mutations may arise only in a subfraction of mtDNA molecules, and consequently, mutant and wild-type mtDNA can co-exist (8). This effect is called heteroplasmy. mtDNA heteroplasmy varies among different human tissues and increases with age, suggesting that the majority of mtDNA heteroplasmies are acquired rather than inherited (9). Research suggests that the progressive accumulation of mutations in the mitochondria over a person’s lifetime may play a central role in aging and in many human diseases.

A relationship that has not been investigated to date is the one between mitochondrial genetic variants and metabolomics (mtGWAS-metabolomics). The most prominent role of mitochondria is not only to generate large quantities of ATP but also to regulate many metabolic tasks (10,11), such as signaling through mitochondrial ROS (12), regulation of the membrane potential (11), apoptosis-programmed cell death (13,14), regulation of cellular metabolism (14), certain heme synthesis reactions (15) and steroid synthesis (16).

The general hypothesis is that an alteration in the concentration of metabolite profiles (possibly conveyed by lifestyle and environmental factors) influences the increase of mutation rate in the mtDNA and thereby contributes to a range of pathophysiological alterations observed in complex diseases. Based on this hypothesis, the rationale for the present research is the establishment of mitochondrial and metabolomic signatures as a panel of candidate biomarkers for the prediction and early diagnosis of disease as well as monitoring of disease progression.

In this study, we conducted association analyses between 151 metabolites and the whole sequenced mitochondrial genome from 1163 individuals to identify genetic variants influencing metabolite profiles. Because of the high coverage, next-generation sequencing (NGS) allows us a deeper and more accurate analysis of the mitochondrial genome for identification of disease-causing variants and heteroplasmy than mitochondrial nucleotide variants (mtSNV) allele intensities measured by genotyping arrays.

**Table 1:** Estimates of the model parameters for the 40 most significant mtSNVs

MT-Gene	Position	mtSNV	Allele	Function	MAF	Metabolite ratio short_name	Beta	*P*-value	Metabolite class
12SrRNA	715	–	G > A	–	–	C2/C10:1	0.909	6.82^*^10^−09^	Acylcarnitine/acylcarnitine
ND1	3714	rs386828920	A > G	Synonymous	0.0002	PC ae C42:5/PC ae C44:5	3.631	1.02^*^10^−08^	Glycerophospholipid/glycerophospholipid
ND4L	10 689	rs879102108	G > A	Missense	0.00139	PC ae C34:2/PC aa C36:6	0.637	1.92^*^10^−08^	Glycerophospholipid/glycerophospholipid
ND4	11 050	rs1603223077	T > C	Synonymous	0.0002	PC aa C34:2/SM:C18:0	2.226	3.62^*^10^−08^	Glycerophospholipid/sphingolipid
ND4L	10 689	rs879102108	G > A	Missense	0.00139	PC ae C36:3/PC aa C36:6	0.637	5.12^*^10^−08^	Glycerophospholipid/glycerophospholipid
ATP6	9071	rs1603222032	C > T	Missense	0.0007	PC aa C34:3/PC aa C32:2	1.286	6.75^*^10^−08^	Glycerophospholipid/glycerophospholipid
ND4	11 050	rs1603223077	T > C	Synonymous	0.0002	PC ae C36:2/SM (OH) C16:1	2.399	6.87^*^10^−08^	Glycerophospholipid/sphingolipid
ND5	12 994	rs1603223993	G > A	Missense	–	PC ae C40:1/PC ae C38:0	1.255	8.57^*^10^−08^	Glycerophospholipid/glycerophospholipid
CYB	15 428	rs1603225270	G > A	Missense	–	PC aa C36:5/PC aa C32:2	0.434	1.01^*^10^−07^	Glycerophospholipid/glycerophospholipid
ND4L	10 645	–	T > C	–	–	SM:C26:0/PC aa C38:5	0.684	1.01^*^10^−07^	Sphingolipid/glycerophospholipid
CO2	7976	rs377368526	G > A	Missense	–	PC ae C36:1/PC aa C36:6	0.839	1.03^*^10^−07^	Glycerophospholipid/glycerophospholipid
CO2	7809	–	T > C	–	–	SM (OH) C14:1/SM:C16:1	1.169	1.33^*^10^−07^	Sphingolipid/sphingolipid
ND4L	10 689	rs879102108	G > A	Missense	0.00139	PC ae C34:3/PC aa C36:6	0.589	1.44^*^10^−07^	Glycerophospholipid/glycerophospholipid
ATP8	8477	rs1603221517	T > C	Missense	0.0007	PC aa C40:6/PC aa C42:2	0.556	1.48^*^10^−07^	Glycerophospholipid/glycerophospholipid
ND5	14 141	–	T > C	–	–	SM (OH) C16:1/C6:1	0.895	1.69^*^10^−07^	Sphingolipid/acylcarnitine
ND4L	10 645	–	T > C	–	–	SM:C26:0/PC aa C36:5	0.478	1.93^*^10^−07^	Sphingolipid/glycerophospholipid
ND4L	10 689	rs879102108	G > A	Missense	0.00139	PC ae C36:1/PC aa C36:6	0.766	2.22^*^10^−07^	Glycerophospholipid/glycerophospholipid
ND4L	10 689	rs879102108	G > A	Missense	0.00139	PC ae C36:2/PC aa C36:6	0.637	2.49^*^10^−07^	Glycerophospholipid/glycerophospholipid
CO1	6207	–	T > C	–	–	SM (OH) C16:1/C5:1-DC	0.744	3.29^*^10^−07^	Sphingolipid/glycerophospholipid
ND4L	10 689	rs879102108	G > A	Missense	0.00139	SM (OH) C14:1/PC aa C36:6	0.62	4.04^*^10^−07^	Sphingolipids/glycerophospholipid
ND4	11 342	–	A > G	–	–	PC ae C40:2/C16:2-OH	0.816	4.22^*^10^−07^	Glycerophospholipid/acylcarnitine
ND4L	10 689	rs879102108	G > A	Missense	0.00139	PC ae C38:3/PC aa C36:6	0.688	4.58^*^10^−07^	Glycerophospholipid/glycerophospholipid
ND4L	10 689	rs879102108	G > A	Missense	0.00139	PC aa C28:1/PC aa C36:6	0.726	4.82^*^10^−07^	Glycerophospholipid/glycerophospholipid
ATP6	9031	rs1556423594	C > T	Synonymous	0.0002	PC ae C42:5/PC ae C44:5	3.21	5.03^*^10^−07^	Glycerophospholipid/glycerophospholipid
ND5	13 356	rs1603224159	T > C	Synonymous	0.0006	C9/lysoPC a C17:0	0.853	5.43^*^10^−07^	Acylcarnitine/glycerophospholipid
ND4	11 088	–	T > C	–	–	C14:1-OH/C16:2-OH	0.849	6.34^*^10^−07^	Acylcarnitine/acylcarnitine
CO1	7279	rs1603220861	T > C	Missense	0.0007	C4/C5:1-DC	0.615	6.36^*^10^−07^	Acylcarnitine/glycerophospholipid
ND4	10 775	rs879015842	G > A	Missense	0.0002	PC aa C32:3/SM (OH) C24:1	1.485	6.51^*^10^−07^	Glycerophospholipid/sphingolipid
tRNA	3241	–	A > G	–	–	PC ae C40:1/PC aa C42:2	0.966	7.30^*^10^−07^	Glycerophospholipid/glycerophospholipid
ND4L	10 689	rs879102108	G > A	Missense	0,00139	PC ae C34:1/PC aa C36:6	0.694	7.37^*^10^−07^	Glycerophospholipid/glycerophospholipid
ND5	14 141	–	T > C	–	–	SM (OH) C16:1/C14:2-OH	0.794	7.60^*^10^−07^	Sphingolipid/acylcarnitine
tRNA	10 031	rs200048690	T > C	–	0.00186	C6 (C4:1-DC)/C16	1.221	7.67^*^10^−07^	Acylcarnitine/acylcarnitine
12SrRNA	856	rs1603218502	A > G	–	0.0007	SM (OH) C16:1/lysoPC a C28:1	0.873	7.76^*^10^−07^	Sphingolipid/glycerophospholipid
CO3	9441	–	C > T	–	–	PC ae C38:4/PC aa C32:0	0.913	8.07^*^10^−07^	Glycerophospholipid/glycerophospholipid
CO1	7115	–	C > T	–	–	PC ae C38:2/PC ae C40:0	1.102	8.10^*^10^−07^	Glycerophospholipid/glycerophospholipid
ND5	12 825	–	T > C	–	–	lysoPC a C14:0/PC aa C38:5	0.762	8.95^*^10^−07^	Glycerophospholipid/glycerophospholipid
CYB	15 373	rs1556424578	A > G	Synonymous	–	PC aa C40:5/PC aa C40:4	2.546	9.36^*^10^−07^	Glycerophospholipid/glycerophospholipid
ND1	3392	–	G > A	–	–	PC aa C40:3/SM:C16:0	1.075	9.72^*^10^−07^	Glycerophospholipid/sphingolipids
ND4	11 493	–	G > A	–	–	PC aa C30:0/PC ae C38:4	0.754	9.78^*^10^−07^	Glycerophospholipid/glycerophospholipid
ND4L	10 689	rs879102108	G > A	Missense	0.00139	PC ae C40:3/PC aa C36:6	0.649	9.92^*^10^−07^	Glycerophospholipid/glycerophospholipid

Legend: Genomic position in base pairs (bp), alleles, rs_number, point mutation, and MAF are based on the NCBI dbSNP GRCh38 human genome assembly (rCRS, GeneBank ID NC_012920.1). Notes: MAF refers to the frequency with which the second most common allele occurs as homoplasmy in the population used by NCBI (how many individuals have only the alternative allele). In the current analysis, MAF was not used, and we used the measure heteroplasmy using individual-level allele frequencies obtained from counts values as proposed by (4). Alleles are given in terms of major > minor allele. Nominal *P*-values are provided for each Beta. mtSNV: mitochondrial single nucleotide variant; MT-Gene: mitochondrialgene.

## Results

We conducted a GWAS with metabolite ratios using 151 metabolic traits in a group of 1163 KORA-F4 individuals with sequenced mitochondria covering 9172 mtSNVs. Linear regression analysis was also conducted for each of 151 metabolites, but the results were by all individual metabolite not significant.

The significant *P*-values are plotted in Figure 2. The *x*-axis represents the mitochondrial genome, showing the position and relative size of each of the 13 major mitochondrial genes. An mtSNV was considered as significant not only when the *P*-value was <1.257545^*^10^−05^ after M_eff_ correction but also when the metabolite ratio *P*-gain was >151, i.e. the total number of metabolites. In total, we observed 404 mtSNVs that display metabolite ratio associations at a genome-wide significance level. Table 1 shows the information of the 40 most significant results.

The most significant mtSNV, mt715_G > A_ located in the MT-12SrRNA, was associated with the metabolite ratio acetylcarnitine/decenoylcarnitine (*P*-value = 6.82^*^10^−9^, *β* = 0.909). Following in significance is mt3714_A > G_ located in the MT-ND1, which was associated with the metabolite ratio phosphatidylcholine (PC) acyl-alkyl C42:5/PC acyl-alkyl C44:5 (*P*-value = 1.02^*^10^−8^, *β* = 3.631).

More details about the estimated model parameters for each significant mtSNV are given in Supplementary Material, Table S1.

It can be observed that a large percentage (15%) of the most significant mtSNVs is located in the MT-ND4L (Fig. 3). They are mostly associated with metabolites from the glycerophospholipid class. The variant mt10689_G > A_, located in the ND4L gene is of special interest because it is associated with 16 different metabolite ratios, making it the most common multi-associated mtSNV in our dataset. Moreover, in all these 16 ratios, the metabolite PC diacyl C36:6 (PC aa C36:6) is involved (Fig. 2).

## Discussion

This study was carried out with the aim of investigating the relationship between the metabolite ratios and the genetic variants of the mtDNA. The general hypothesis is that an alteration in the concentration of metabolite profiles (possibly conveyed by lifestyle and environmental factors) influences the increase of mutation rate in the mtDNA and thereby contributes to a range of pathophysiological alterations observed in complex diseases.

### Decenoylcarnitine/acetylcarnitine associated with MT-12SrRNA_mt715G > A_ gene

The strongest association was found for mt715_G > A_ located in the MT-12SrRNA with the metabolite ratio acetylcarnitine (C2)/decenoylcarnitine (C10:1) (*P*-value = 6.82^*^10^−9^, *β* = 0.909). This association may play a crucial role in the regulation of insulin secretion.

C2 is broken down in the blood by plasma esterases to carnitine which is used by the body to transport fatty acids into the mitochondria for breakdown and thus may have a role in normalizing intracellular lipid metabolism. It is known that C2 levels are low in patients with type 1 and type 2 diabetes mellitus (17). Furthermore, therapy with C2 ameliorated arterial hypertension, insulin resistance, impaired glucose tolerance and hypoadiponectinemia in subjects at increased cardiovascular risk (18). It has also been reported that oral administration of acetylcarnitine increases insulin sensitivity and glucose tolerance in individuals with a low glucose disposal rate (19).

C10:1 is considered to be a fatty ester lipid molecule. This metabolite was found to be associated not only with type 2 diabetes but also with prediabetic states (20). In addition, C10:1 is used in the diagnosis of genetic disorders, such as fatty acid oxidation disorders (21), in which insulin plays a crucialrole.

In humans, MT-12SrRNA is encoded by the MT-RNR1 gene. The MT-RNR1 gene encodes for a protein responsible for regulating insulin sensitivity and metabolic homeostasis (22). The protein acts as an inhibitor of the folate cycle, thereby reducing *de novo* purine biosynthesis, which leads to the accumulation of the *de novo* purine synthesis intermediate 5-aminoimidazole-4-carboxamide and the activation of the metabolic regulator 5′-AMP-activated protein kinase.

Interestingly, the concentration of C2 and C10:1 has been observed to increase during physical exercise (23,24). It is well known that insulin levels typically decrease during exercise.

In this context, our results show that an alteration of C2/C10:1 levels in serum increases the ratio of heteroplasmy at mt715_G > A_ which, in turn, can lead to MT-12SrRNA dysfunction, possibly even to affect insulin regulation.

### Phosphatidylcholine acyl-alkyl C42:5 (PC ae C42:5)/PC acyl-alkyl C44:5 (PC ae C44:5) is associated with the MT-ND1_mt3714A > G_ gene

The second most significant mtSNV was found for mt3714_A > G_ located in the MT-ND1 with the metabolite ratio PC ae C42:5/PC ae C44:5 (*P*-value = 1.02^*^10^−08^, *β* = 3.631). This association may play a crucial role in disorders related to damage or disease that affects the brain.

Both metabolites involved in this ratio are PC, which are included in the glycerophospholipid class. Glycerophospholipids are the main component of biological membranes, providing them with stability, fluidity and permeability. Glycerophospholipids are actively catabolized by brain tissue and are involved in the regulation of several molecular functions, such as generation of second messengers, apoptosis, antioxidant and membrane fusion and regulation of enzyme activities (25). A marked deficit in glycerophospholipid concentration may be responsible for the neurodegeneration observed in neurological disorders (26). In particular, alteration in PC metabolism has been observed in Alzheimer, Parkinson and with an increased risk of dementia (27–29). Several studies have used dietary intake of PC in clinical trials treating brain diseases, resulting with an improvement of the memory, capability of learning and cognitive performances (28,30,31). Recently, supplementation with PC has been also associated with a reduced risk of dementia (29).

The MT-ND1 gene encodes for the NADH–ubiquinone oxidoreductase chain 1 protein, a subunit of NADH dehydrogenase, which is located in the mitochondrial inner membrane. Genetic mutations of the human MT-ND1 gene have been associated with several inborn genetic disorders, such as MELAS, Leigh’s syndrome and Leber’s hereditary optic neuropathy (32,33). All these inborn disorders are related to damage or disease that affects the brain. Also, a decreased DNA methylation has been observed in the MT-ND1 gene in cases with early stage of Alzheimer’s disease (34).

Based on our results, an alteration in PC ae C42:5 and PC ae C44:5 increases heteroplasmy at mt3714_A > G_ which, in turn, can lead to MT-ND1 dysfunction. This represents a pathway that may help to understand molecular aspects of neurodegeneration diseases.

### MT-ND4L—mitochondrially encoded NADH: ubiquinone oxidoreductase core subunit 4L

It should be noted that the largest number of significant associations between metabolite ratios and mtSNVs are located in the MT-ND4L gene (Figs 2 and 3). The variant mt10689_G > A_, located in the ND4L gene, which is associated with 16 metabolite ratios is the most common multi-associated mtSNV in our dataset. Moreover, in all these 16 ratios, the metabolite PC diacyl C36:6 (PC aa C36:6) is involved.

Despite the crucial importance of PC for brain functioning, disturbance of PC concentration has also been found to be associated with various metabolic disorders, such as atherosclerosis (35), insulin resistance (35,36), Gaucher disease (37) and obesity in adults (38,39) as well as in children (40). Particularly, the metabolite PC aa C36:6 has been associated with different patterns of fat concentration in the body, such as visceral fat and liver fat content (41).

PC aa C36:6 was also involved in three metabolite ratios that were previously shown to be associated with Fat-Free Mass Index (42).

MT-ND4L is a subunit of NADH dehydrogenase, which is the component of the electron transport chain that is responsible for the oxidative phosphorylation process. Its dysfunction may cause energy deficiency in cells, resulting in metabolic disorders such as obesity and diabetes (43–45). In fact, several variants of human MT-ND4L have been reported to be associated with altered metabolic conditions like BMI and type 2 diabetes (43,46). Changes in MT-ND4L gene expression have long-term consequences on energy metabolism and have been suggested to be a major predisposition factor for the development of metabolic syndrome (47).

Based on our results in 16 metabolite ratios where PC aa C36:6 is involved, alteration of its concentration changes significantly the heteroplasmy in the variant mt10689_G > A_, located in the ND4L gene. Put together, this association may explain an interesting pathway to understand the development of metabolic conditions.

## Conclusion

We presented the results of the first association analysis between genetic positions in the mitochondrial genome and metabolite profiles using sequencing data of the mitochondrial genome and an inverted mtGWAS. Although further analyses are needed to follow up on the present results, these findings highlight the important role of the mtDNA and the field of metabolomics among the factors that contribute to the balance of the human metabolism and suggest that variants in the mitochondrial genome may be more important than has previously been suspected.

Our results support the idea that a strong relationship exists between mtDNA heteroplasmy and metabolite levels. Both are likely to play a crucial role as discriminating cofactors in the etiology of common multi-factorial diseases. A conceivable hypothesis is that high levels of oxidative stress might be produced when altered levels of certain metabolites (possibly owing to environmental factors such as nutrition or life style) alter the permeability of cell membranes along with increasing the heteroplasmic mtDNA and weakening the mitochondrial defense systems. High levels of oxidative stress damage cellular structures, including the mitochondria themselves, which, in turn, can lead to mitochondrial dysfunction and possibly even to apoptosis.

In the era of personalized medicine with more and more patient-specific targeted therapies being used, we need reliable, dynamic, faster and sensitive biomarkers both to track the causes of disease and to develop and evolve therapies during the course of treatment. Understanding the mechanisms that control human health and disease, in particular, the role of genetic predispositions and their interaction with environmental factors, is a prerequisite for the development of safe and efficient therapies for complex disorders.

We conclude that recent advances in metabolomics and NGS technology along with novel strategies to analyze and understand the metabolic pathways, as well as to integrate metabolite networks with mitochondrial genetic data, opens this window of opportunity to identify new biomarkers related to the human mtDNA, both to track complex diseases and to develop and evolve the option of treatment.

## Materials and Methods

### Study design and population

The Cooperative Health Research in the Region of Augsburg (KORA) study is a series of independent population-based epidemiological surveys and follow-up studies of participants living in the region of Augsburg, in southern Germany, an area with demographic and socioeconomic characteristics roughly reflecting those of an average central European population. All participants are residents of German nationality who were identified through the registration office, and written informed consent was obtained from each participant (48). The study was approved by the local ethics committee (Bayerische Landesärztekammer). All participants filled in a self-administrated questionnaire and underwent a standardized personal interview and an extensive medical examination. Detailed phenotypes and personal information related to life circumstances, history of disease and medication were recorded in a computer-assisted personal interview. All procedures were subjected to quality assessment. The study design, sampling method and data collection have been described in detail elsewhere (49). The participants of this study were selected from the KORA-F4 (2006–2008) study, including a total number of 3021 unrelated individuals. KORA-F4 is the first follow-up study of the population-based survey KORA S4 (1999–2001). All procedures were subjected to quality assessment. No evidence of population stratification has been found in multiple published analyses using genetic data of the KORA cohort.

### Metabolite profiles

Metabolomic measurements have been performed for 3061 individuals of KORA F4 population-based sample. Men and women were collected in a random order and samples were randomly put on plates to reduce batch effects. Metabolites were measured in serum using the AbsoluteIDQ™ p150 Kit (BIOCRATES Life Sciences AG, Innsbruck, Austria), as described elsewhere (3). The panel includes 151 metabolites spanning several metabolic classes: 1 hexose, 35 acylcarnitines, 14 amino acids, 14 sphingomyelins and 87 glycerophospholipids.

### Sequencing of the mitochondrial genome

The mitochondrial genome was sequenced for 3021 KORA-F4 individuals using DNA derived from peripheral blood mononuclear cells. A long-range PCR approach, which refers to the amplification of DNA fragments of a size that may not be amplified using conventional PCR reagents, has been performed using a highly processive polymerase mixture and novel primer pairs to specifically amplify the mitochondrial genome (50). These samples were subsequently processed with Illumina^®^ Nextera^®^ XT (catalog # FC-131-1096). This NGS library preparation kit employs an engineered Transposome™ to randomly fragment and tag amplicons and small genomes with Illumina^®^ specific adapters. After library preparation, samples were sequenced on the Illumina^®^ MiSeq™. This method generated whole mitochondrial genome NGS data, which accurately reflect the Sanger sequence. The 16 569 positions have been sequenced with mtSNV covered with 3500-fold on average. Because of this high coverage, NGS will allow us a much deeper analysis of the mitochondrial genome and heteroplasmy for identification of disease-causing variants than array-based genotyping of mitochondrial SNVs. It has been observed that NGS data are highly reproducible and very reliable, and, overall, they has been found to be superior to the data produced by microarray technology (51).

### Quality control

**Figure 1:**
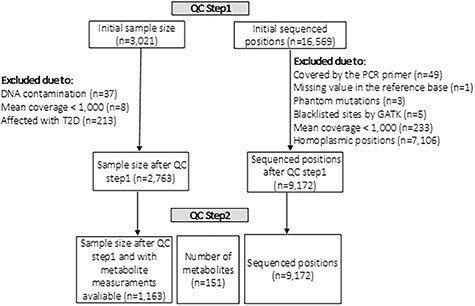
Schema of the quality control performed in the datasets.

Before analyzing the data, we performed a two-step quality control. In QC step1, we checked the sequencing data, while in QC step2, we focused on the quality of the metabolites (see Fig. 1).

During QC step1, a total number of 258 out of 3021 individuals were excluded. Thirty-seven individuals were excluded owing to DNA contamination and eight individuals were excluded because of an overall mean coverage <1000. In order to avoid confounding with insulin-dependent diabetes mellitus, 213 individuals who were diagnosed with type 2 diabetes were not included in the study. The mitochondrial region covered by the PCR primers spanning 49 bases, from position 16 401 to 16 449, had to be excluded from the analysis as it does not contain any reliable information. Position mt3107 was removed owing to its missing value in the reference base. In addition, the three known phantom mutations (mt3166, mt3170 and mt16390) were also removed together with the five positions (mt301, mt302, mt310, mt316 and mt16182) in blacklisted sites as recommended by GATK (52). Further 233 positions were excluded because of mean coverage <1000. As we were primarily interested in somatic mtDNA variants, 7106 homoplasmic positions were also excluded since, for association analysis, they do not provide any type of information. The threshold to call a position as homoplasmic for the reference allele was 0.007(see Supplementary Material S1). Afterward, we searched for the presence of nuclear-mitochondrial-DNA fragments (NUMTS) using mtDNA-server (53). Because NUMTS are copies of mtDNA sequences inserted in the nDNA over evolutionary time, these sequences are possible handicaps for data processing, as the sequenced samples might contain some nDNA fragments. If they are falsely counted as mtDNA fragments, the allele frequencies could be erroneous, and consequently, incorrect heteroplasmy values would be determined. We identified a total number of 343 NUMTS. However, in our case, a PCR with primers specific to the mtDNA has been performed in the beginning and so it is very unlikely that the sequenced fragments contain nDNA. Therefore, these NUMTS do not need to be removed from the analysis because they are adding useful information. After QC step1, a total number of 9172 mtSNVs and 2763 individuals were considered for QC step2.

**Figure 2:**
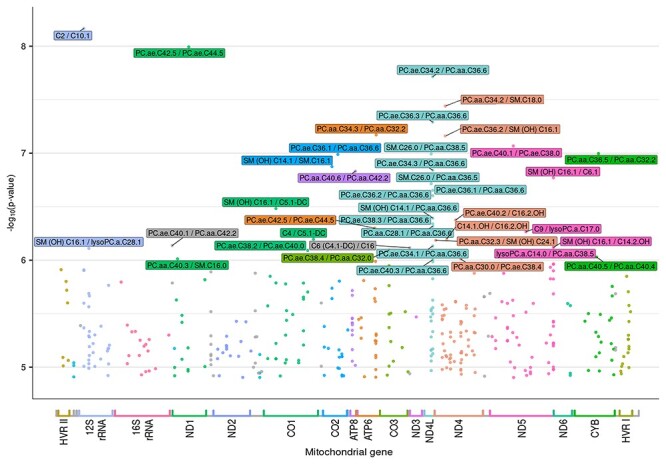
Mitochondrial genome-wide *P*-values for metabolite ratios. Legend: On the *y* axis, *P*-values transformed into the negative of the base 10 logarithm, −log10(*P*-value), are shown. The *x*-axis represents the mitochondrial genome, displaying the position and relative size of each of the 13 major mitochondrial genes, 12S and 16S rRNAs, hypervariable region 1 (HVR I), hypervariable region 2 (HVR II) as well as the position of the 22 tRNAs (gray color).

**Figure 3:**
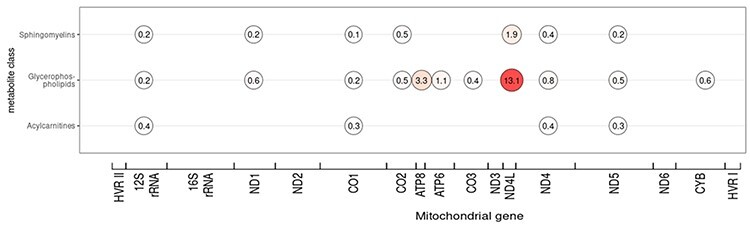
The distribution of significant metabolites for metabolite classes across mitochondrial genes. Legend: Given a mitochondrial gene, a circle shows the percentage of metabolites involved in significant ratios with regard to their metabolite class. The 40 most significant mtSNVs are considered. Because the genes have different lengths, the number of mtSNVs tested in each gene is different. The percentage has been calculated by taking the number of significant metabolite ratios divided by the total number of mtSNVs tested for eachgene.

During QC step2, metabolites were imputed using multiple imputation by chained equations with the method called ‘predictive mean matching’. Missing values are frequently observed in high-throughput, mass spectrometry-based metabolomics measurements, which is the approach used by Biocrates. To this end, we have used the same approach as in previous analyses of the Biocrates metabolomics data in KORA (2,36). Following the criteria by (54) to avoid spurious false-positive associations owing to small sample sizes, at least 80% of non-missing values are required for a metabolite to be included, and data points of metabolic traits lying more than three standard deviations off the mean need to be excluded. All of the 151 available metabolites satisfied this criterion.

After quality control, individuals with available mtDNA data and metabolite values were considered in the analysis. Association analyses was performed in a total number of 9172 mtSNVs and 151 metabolites in a sample of 1163 KORA-F4 individuals.

### Statistical methods

GWAS have been developed as a method to identify genetic loci associated with disease by scanning multiple markers across the genome. The advance in genotyping technology has led to significant advances in the genetics of complex diseases. Recently, NGS has gained popularity through its capacity to analyze a much greater number of markers across the genome. One of the peculiarities of the mtDNA is the heteroplasmy. Owing to heteroplasmy, mtDNA tends to be heterogeneous in the sense that different mitochondria can have different genotypes such that a genotype at an mtDNA locus may not be restricted to zero, one or two minor alleles. This issue affects the possibility of estimating genotypes and makes calling algorithms useless. The finest way to approach mitochondrial heteroplasmy is to utilize high-throughput sequencing data, which readily provides data of the entire mitochondrial genome and offers the prerequisite for the detection of low-level mitochondrial genetic heteroplasmy.

In our analysis, we take the heteroplasmy into account by using the raw sequence reads, i.e. the number of counts for each variant, instead of calling the variants into a few categories. The best way to assess the association of metabolites with the mtSNV is to apply linear regression analysis. The mtSNV enters the model as the response via the log_2_-transformed ratio, *y* = log_2_(B/A), where A denotes the number of counts of the reference allele and B the second most frequent allele. In the ratios, to avoid undefined logarithms in those individuals who only carry the reference allele for the mtSNV under study, a term of two additional counts was added in the denominator and numerator. Considering that the average number of counts is about 3500, adding two counts to A and B will not have any impact in the final results.

Sex and age at examination are introduced in the model as covariates. To improve the convergence properties of model estimates, the total coverage (Cov) of the mtSNV (i.e. A + B) was added in the model. In addition, to avoid false positives owing to a batch effect during mtDNA sequencing, the effect of the index of the sequencing plate (Batch_mtDNA_) was included in the model aswell.

It has been suggested several times that the use of ratios of two metabolite concentrations, which serve as proxies for enzymatic reaction rates, not merely reduces the variance and increases the power of GWAS, thus yielding robust statistical associations, but as well provides new information about possible metabolic pathways (2,54,55). Therefore, we considered all possible metabolite concentration ratios (MR_1_, … MR_(N^*^(N-1))/2_), where N is the number of single metabolites, with the linear model for association analysis with all mtSNVs. This hypothesis-free approach highlights pairs of metabolites that are more likely to be coupled either biochemically or physiologically (3).

The regression model for the *j-*th metabolite ratio and the *i-*th mtSNV is

$$\begin{array}{l} {y}_{ik}={\beta}_0+{\beta}_1{\mathrm{MR}}_{\mathrm{jk}}+{\beta}_2{\mathrm{age}}_{\mathrm{k}}+{\beta}_3{\mathrm{sex}}_{\mathrm{k}}+{\beta}_4{\mathrm{Cov}}_{\mathrm{ik}}\\+{\beta}_5{\left({\mathrm{Batch}}_{\mathrm{mtDNA}}\right)}_k+{e}_{ijk} \end{array}$$

for *j* = 1,…,*m*; *i* = 1,…*s*; *k* = 1,…*n*,

where *k* denotes the individual and *n* is the sample size, *m* the number of metabolite ratios (N^*^(*N* − 1))/2) and *s* the number of mtSNVs to be tested. The aim of this analysis is to evaluate how the mitochondrial heteroplasmy is influenced by the metabolite ratio, representing the phenotype of interest, taking into account the age and sex of a particular person; and therefore, the model adjusts for individual differences regarding these two variables in the study population. GWAS has always been referred to analyses that use genetic variance as predictors in models. In our analysis, the general hypothesis is that an alteration in the concentration of metabolite profiles influences the increase of mutation rate in the mtDNA. In this situation, it is more appropriate to use the genetic variants as outcome variables. For that reason, we will refer to this approach as ‘inverted mtGWAS’.

### Correction for multiple testing

*P*-values are obtained from a Wald test based on the asymptotic normality of regression coefficient estimates and are corrected for multiple comparisons, with the correction factor being equal to the effective number of independent tests (*M*_eff_) (56). We calculated the *M*_eff_ measure using the Matrix Spectral Decomposition (matSpDlite) from Nyholt *et al*. (57) with the method of Li and Ji (58). We used the *P*-gain statistics (2) to quantify the decrease in *P*-value for the association with the ratio compared with the *P*-values of the two corresponding concentrations. This limit is considered a Bonferroni-type conservative cutoff for identifying whether a ratio between two metabolite concentrations improves the strength of association compared with the two corresponding metabolite concentrations alone. In our case, *P*-gain should be larger than the number of tested metabolic traits (*P*-gain > 151). To estimate whether deviation from normality of metabolite ratios may have biased our results, we tested associations for both untransformed and log-scaled ratios, not detecting substantial differences. Quantile-quantile plots were used to examine the *P*-value distribution, and the lambda (*λ*) ranged from 0.93 to 1.26 (see Supplementary Material, Fig. S1). All statistical analyses were performed using RStudio version 0.98.1103 that uses R version 3.5.2 (59).

## Supplementary Material

supplementary_Figure_1_ddab312

supplementary_Material_1_ddab312

supplementary_Table1_ddab312
