# Supplementary figures and images for: First mitochondrial genome-wide association study with metabolomics
Source: Hum Mol Genet. 2021 Oct 27;31(19):3367–76. doi: 10.1093/hmg/ddab312 (PMC9523559; doi:10.1093/hmg/ddab312)

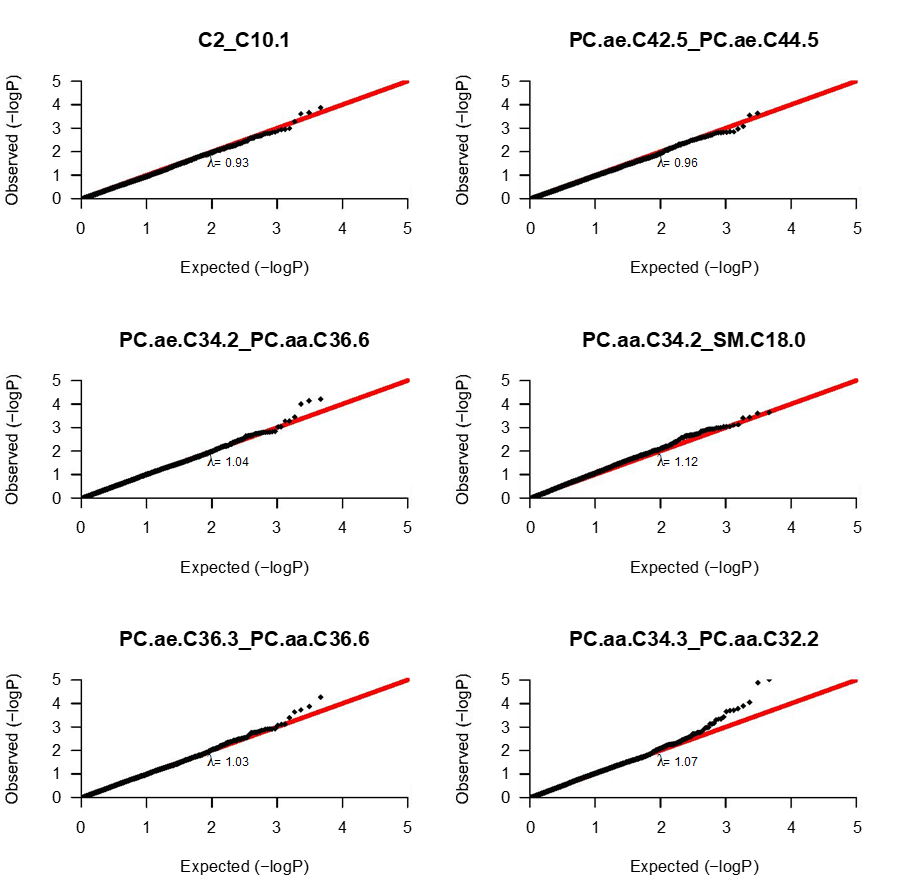


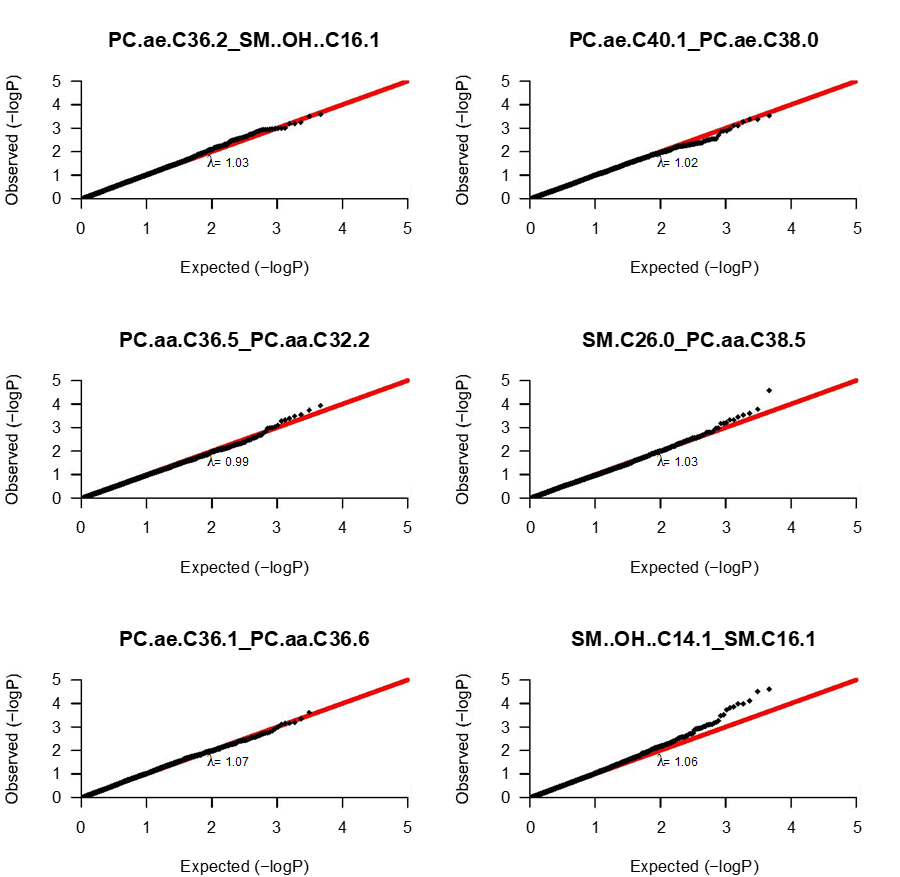


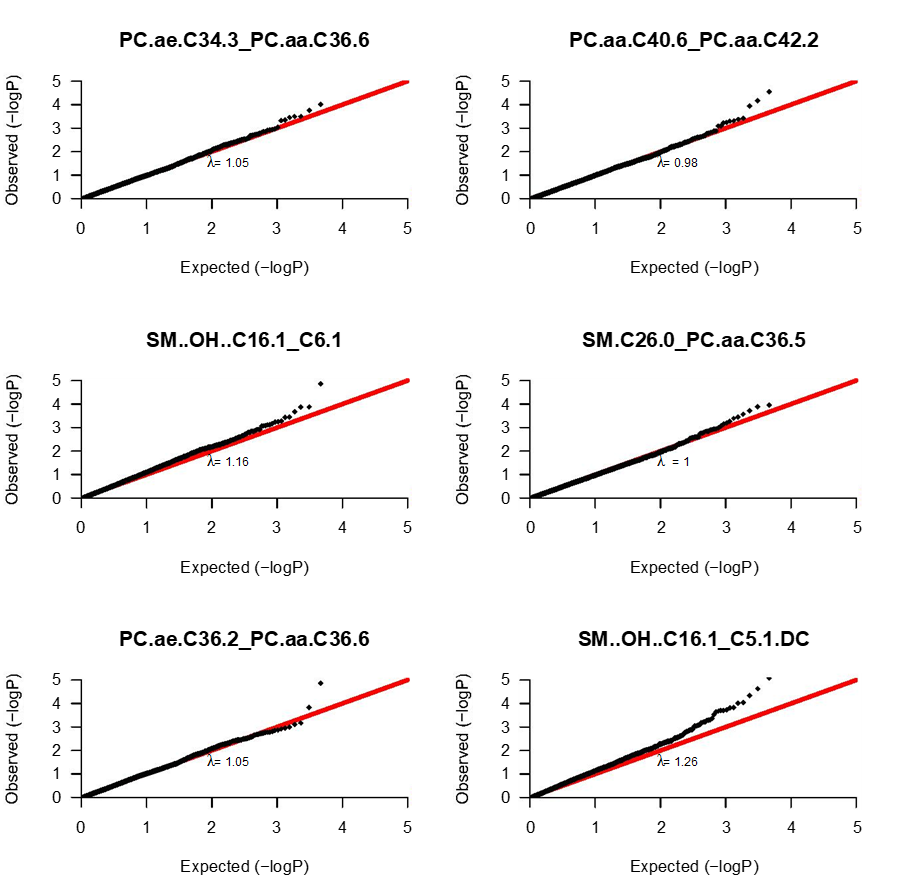


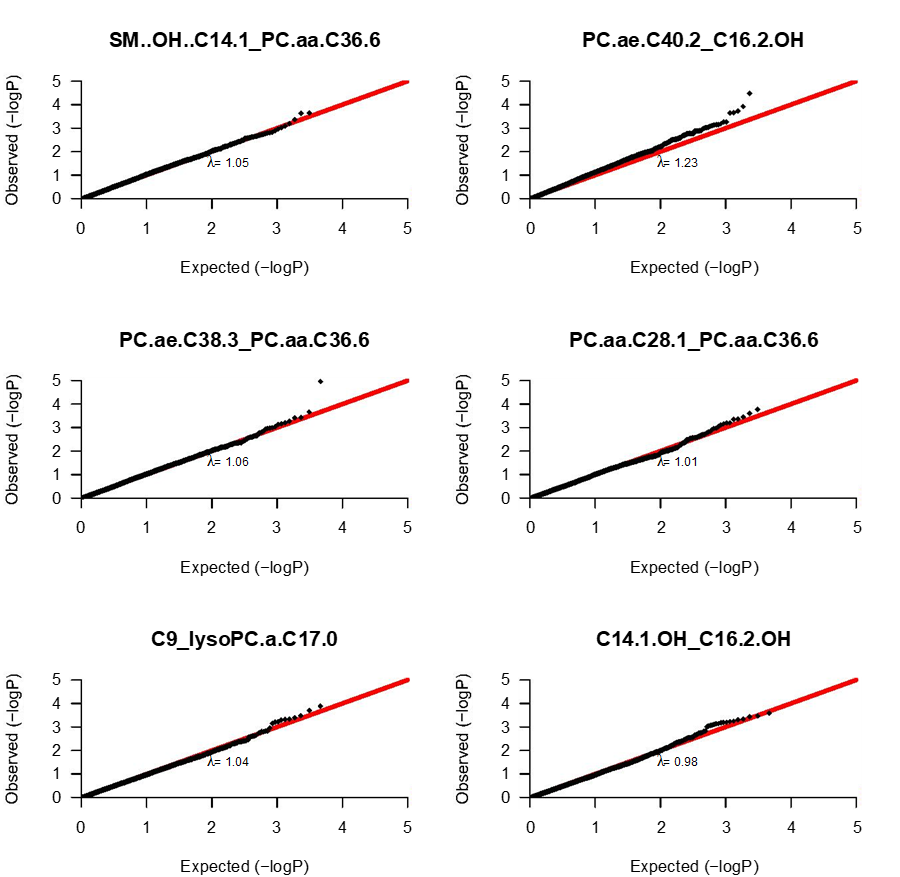


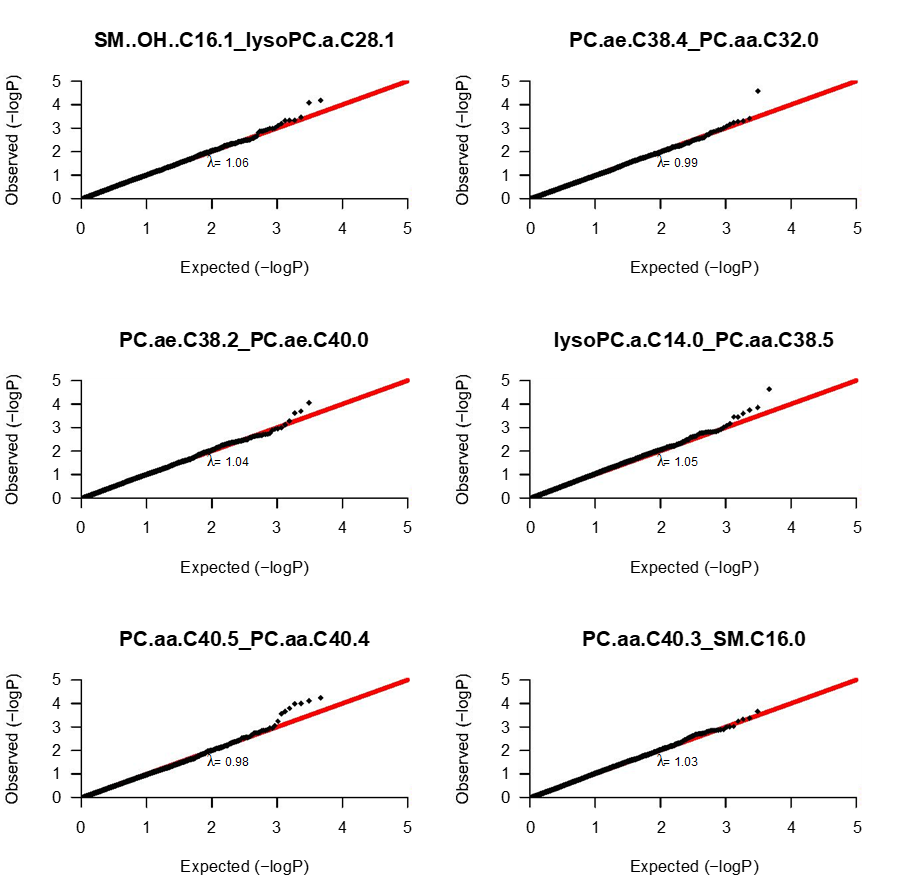


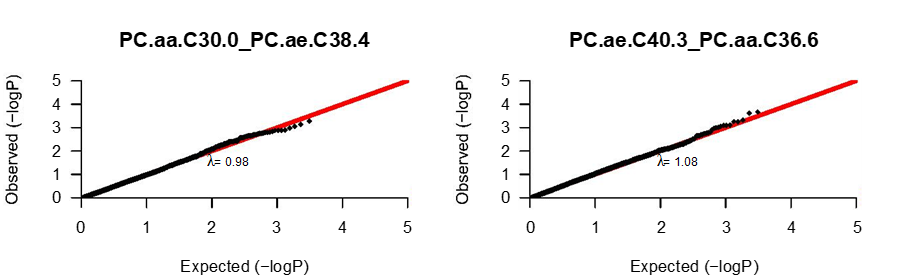


Figure S1: Q-Q plot for the significant association tests

Supplement: supplementary_Figure_1_ddab312 [file supplementary_figure_1_ddab312.docx]
