# Supplementary material for: First mitochondrial genome-wide association study with metabolomics
Source: Hum Mol Genet. 2021 Oct 27;31(19):3367–76. doi: 10.1093/hmg/ddab312 (PMC9523559; doi:10.1093/hmg/ddab312)
Supplement: supplementary_Material_1_ddab312 [file supplementary_material_1_ddab312.docx]

**Selection of the Heteroplasmy threshold**

The threshold to call a position as homoplastic for the reference allele was 0.007. This threshold was selected as follows:

1. First of all for each position we computed the average heteroplasmy, i.e., proportion of alternative allele, over 3,021 individuals.
2. Positions with a low average were classified in one of the 4 groups according to their average heteroplasmy: <0.005, [0.005,0.01), [0.01,0.05), and [0.05,0.1).
3. 40 positions of each group were randomly selected and plotted. Here is an example **of one position** of each group:

A visual check was performed of all plots. It was clear that in the first group there was very low variability. The variability started to be apparent from the second group.


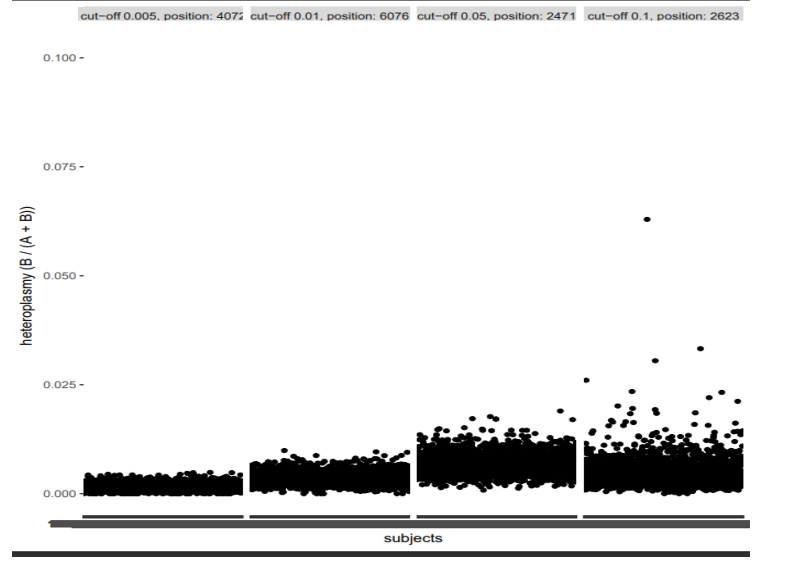


1. Next, we focused on additional cut-offs in the range between 0.005 and 0.01, i.e., we chose 40 positions with a mean heteroplasmy proportion lower than 0.005, [0.005,0.006), [0.006,0.007), and [0.007,0.01). One example for each of these categories is shown in the next figure:


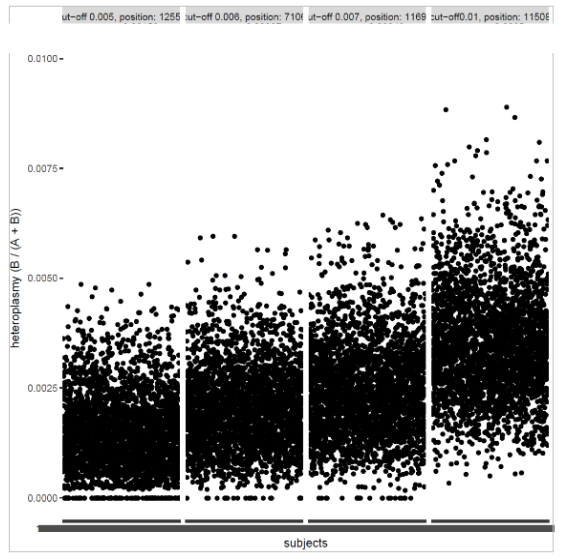


1. We performed a second visual view and the largest difference was in most of the plots between 0.007 and 0.01, an example for which is shown in the right most panel of the plot. According to these plots, we defined the cut-off as 0.007 for differentiating between homoplasmic and heteroplasmic positions.
