# Supplementary material for: First mitochondrial genome-wide association study with metabolomics
Source: Hum Mol Genet. 2021 Oct 27;31(19):3367–76. doi: 10.1093/hmg/ddab312 (PMC9523559; doi:10.1093/hmg/ddab312)
Supplement: supplementary_Table1_ddab312 [file supplementary_table1_ddab312.docx]

Table 1. **Estimates of the model parameters for mtSNPs**

| **MT-Gene** | **Position** | **mtSNP** | **Allele** | **Function** | **Metabolite ratio Biochemical Name** | **Metabolite ratio short_name** | **Beta** | **p-value** | **Metabolite class** |
| --- | --- | --- | --- | --- | --- | --- | --- | --- | --- |
| 12SrRNA | 715 | - | G > A | - | Acetyl-L-carnitine / Decenoyl-L-carnitine | C2 / C10:1 | 0.909 | 6.82*10^-09^ | Acylcarnitine / Acylcarnitine |
| ND1 | 3714 | rs386828920 | A > G | synonymous | PhosphatidylCholine acyl-alkyl C42:5 / PhosphatidylCholine acyl-alkyl C44:5 | PC ae C42:5 / PC ae C44:5 | 3.631 | 1.02*10^-08^ | Glycerophospholipid / Glycerophospholipid |
| ND4L | 10689 | rs879102108 | G > A | missense | PhosphatidylCholine acyl-alkyl C34:2 / PhosphatidylCholine diacyl C36:6 | PC ae C34:2 / PC aa C36:6 | 0.637 | 1.92*10^-08^ | Glycerophospholipid / Glycerophospholipid |
| ND4 | 11050 | rs1603223077 | T > C | synonymous | PhosphatidylCholine diacyl C34:2 / Shingomyeline C 18:0 | PC aa C34:2 / SM C18:0 | 2.226 | 3.62*10^-08^ | Glycerophospholipid / Sphingolipid |
| ND4L | 10689 | rs879102108 | G > A | missense | PhosphatidylCholine acyl-alkyl C36:3 / PhosphatidylCholine diacyl C36:6 | PC ae C36:3 / PC aa C36:6 | 0.637 | 5.12*10^-08^ | Glycerophospholipid / Glycerophospholipid |
| ATP6 | 9071 | rs1603222032 | C > T | missense | PhosphatidylCholine diacyl C34:3 / PhosphatidylCholine diacyl C32:2 | PC aa C34:3 / PC aa C32:2 | 1.286 | 6.75*10^-08^ | Glycerophospholipid / Glycerophospholipid |
| ND4 | 11050 | rs1603223077 | T > C | synonymous | PhosphatidylCholine acyl-alkyl C36:2 / Hydroxyshingomyeline C 16:1 | PC ae C36:2 / SM (OH) C16:1 | 2.399 | 6.87*10^-08^ | Glycerophospholipid / Sphingolipid |
| ND5 | 12994 | rs1603223993 | G > A | missense | PhosphatidylCholine acyl-alkyl C40:1 / PhosphatidylCholine acyl-alkyl C38:0 | PC ae C40:1 / PC ae C38:0 | 1.255 | 8.57*10^-08^ | Glycerophospholipid / Glycerophospholipid |
| CYB | 15428 | rs1603225270 | G > A | missense | PhosphatidylCholine diacyl C36:5 / PhosphatidylCholine diacyl C32:2 | PC aa C36:5 / PC aa C32:2 | 0.434 | 1.01*10^-07^ | Glycerophospholipid / Glycerophospholipid |
| ND4L | 10645 | - | T > C | - | Shingomyeline C 26:0 / PhosphatidylCholine diacyl C38:5 | SM C26:0 / PC aa C38:5 | 0.684 | 1.01*10^-07^ | Sphingolipid / Glycerophospholipid |
| CO2 | 7976 | rs377368526 | G > A | missense | PhosphatidylCholine acyl-alkyl C36:1 / PhosphatidylCholine diacyl C36:6 | PC ae C36:1 / PC aa C36:6 | 0.839 | 1.03*10^-07^ | Glycerophospholipid / Glycerophospholipid |
| CO2 | 7809 | - | T > C | - | Hydroxyshingomyeline C 14:1 / Shingomyeline C 16:1 | SM (OH) C14:1 / SM C16:1 | 1.169 | 1.33*10^-07^ | Sphingolipid / Sphingolipid |
| ND4L | 10689 | rs879102108 | G > A | missense | PhosphatidylCholine acyl-alkyl C34:3 / PhosphatidylCholine diacyl C36:6 | PC ae C34:3 / PC aa C36:6 | 0.589 | 1.44*10^-07^ | Glycerophospholipid / Glycerophospholipid |
| ATP8 | 8477 | rs1603221517 | T > C | missense | PhosphatidylCholine diacyl C40:6 / PhosphatidylCholine diacyl C42:2 | PC aa C40:6 / PC aa C42:2 | 0.556 | 1.48*10^-07^ | Glycerophospholipid / Glycerophospholipid |
| ND5 | 14141 | - | T > C | - | Hydroxyshingomyeline C 16:1 / Hexenoyl-L-carnitine | SM (OH) C16:1 / C6:1 | 0.895 | 1.69*10^-07^ | Sphingolipid / Acylcarnitine |
| ND4L | 10645 | - | T > C | - | Shingomyeline C 26:0 / PhosphatidylCholine diacyl C36:5 | SM C26:0 / PC aa C36:5 | 0.478 | 1.93*10^-07^ | Sphingolipid / Glycerophospholipid |
| ND4L | 10689 | rs879102108 | G > A | missense | PhosphatidylCholine acyl-alkyl C36:1 / PhosphatidylCholine diacyl C36:6 | PC ae C36:1 / PC aa C36:6 | 0.766 | 2.22*10^-07^ | Glycerophospholipid / Glycerophospholipid |
| ND4L | 10689 | rs879102108 | G > A | missense | PhosphatidylCholine acyl-alkyl C36:2 / PhosphatidylCholine diacyl C36:6 | PC ae C36:2 / PC aa C36:6 | 0.637 | 2.49*10^-07^ | Glycerophospholipid / Glycerophospholipid |
| CO1 | 6207 | - | T > C | - | Hydroxyshingomyeline C 16:1 / Glutaconyl-L-carnitine | SM (OH) C16:1 / C5:1-DC | 0.744 | 3.29*10^-07^ | Sphingolipid / Glycerophospholipid |
| ND4L | 10689 | rs879102108 | G > A | missense | Hydroxyshingomyeline C 14:1 / PhosphatidylCholine diacyl C36:6 | SM (OH) C14:1 / PC aa C36:6 | 0.62 | 4.04*10^-07^ | Sphingolipid / Glycerophospholipid |
| ND4 | 11342 | - | A > G | - | PhosphatidylCholine acyl-alkyl C40:2 / Hydroxyhexadecadienyl-L-carnitine | PC ae C40:2 / C16:2-OH | 0.816 | 4.22*10^-07^ | Glycerophospholipid / Acylcarnitine |
| ND4L | 10689 | rs879102108 | G > A | missense | PhosphatidylCholine acyl-alkyl C38:3 / PhosphatidylCholine diacyl C36:6 | PC ae C38:3 / PC aa C36:6 | 0.688 | 4.58*10^-07^ | Glycerophospholipid / Glycerophospholipid |
| ND4L | 10689 | rs879102108 | G > A | missense | PhosphatidylCholine diacyl C28:1 / PhosphatidylCholine diacyl C36:6 | PC aa C28:1 / PC aa C36:6 | 0.726 | 4.82*10^-07^ | Glycerophospholipid / Glycerophospholipid |
| ATP6 | 9031 | rs1556423594 | C > T | synonymous | PhosphatidylCholine acyl-alkyl C42:5 / PhosphatidylCholine acyl-alkyl C44:5 | PC ae C42:5 / PC ae C44:5 | 3.21 | 5.03*10^-07^ | Glycerophospholipid / Glycerophospholipid |
| ND5 | 13356 | rs1603224159 | T > C | synonymous | Nonayl-L-carnitine / lysoPhosphatidylCholine acyl C17:0 | C9 / lysoPC a C17:0 | 0.853 | 5.43*10^-07^ | Acylcarnitine / Glycerophospholipid |
| ND4 | 11088 | - | T > C | - | Hydroxytetradecenoyl-L-carnitine / Hydroxyhexadecadienyl-L-carnitine | C14:1-OH / C16:2-OH | 0.849 | 6.34*10^-07^ | Acylcarnitine / Acylcarnitine |
| CO1 | 7279 | rs1603220861 | T > C | missense | Butyryl-L-carnitine / Glutaconyl-L-carnitine | C4 / C5:1-DC | 0.615 | 6.36*10^-07^ | Acylcarnitine / Glycerophospholipid |
| ND4 | 10775 | rs879015842 | G > A | missense | PhosphatidylCholine diacyl C32:3 / Hydroxyshingomyeline C 24:1 | PC aa C32:3 / SM (OH) C24:1 | 1.485 | 6.51*10^-07^ | Glycerophospholipid / Sphingolipid |
| tRNA | 3241 | - | A > G | - | PhosphatidylCholine acyl-alkyl C40:1 / PhosphatidylCholine diacyl C42:2 | PC ae C40:1 / PC aa C42:2 | 0.966 | 7.30*10^-07^ | Glycerophospholipid / Glycerophospholipid |
| ND4L | 10689 | rs879102108 | G > A | missense | PhosphatidylCholine acyl-alkyl C34:1 / PhosphatidylCholine diacyl C36:6 | PC ae C34:1 / PC aa C36:6 | 0.694 | 7.37*10^-07^ | Glycerophospholipid / Glycerophospholipid |
| ND5 | 14141 | - | T > C | - | Hydroxyshingomyeline C 16:1 / Hydroxytetradecadienyl-L-carnitine | SM (OH) C16:1 / C14:2-OH | 0.794 | 7.60*10^-07^ | Sphingolipid / Acylcarnitine |
| tRNA | 10031 | rs200048690 | T > C | - | Fumaryl-L-carnitine / Hexanoyl-L-carnitine / Hexadecanoyl-L-carnitine | C6 (C4:1-DC) / C16 | 1.221 | 7.67*10^-07^ | Acylcarnitine / Acylcarnitine |
| 12SrRNA | 856 | rs1603218502 | A > G | - | Hydroxyshingomyeline C 16:1 / lysoPhosphatidylCholine acyl C28:1 | SM (OH) C16:1 / lysoPC a C28:1 | 0.873 | 7.76*10^-07^ | Sphingolipid / Glycerophospholipid |
| CO3 | 9441 | - | C > T | - | PhosphatidylCholine acyl-alkyl C38:4 / PhosphatidylCholine diacyl C32:0 | PC ae C38:4 / PC aa C32:0 | 0.913 | 8.07*10^-07^ | Glycerophospholipid / Glycerophospholipid |
| CO1 | 7115 | - | C > T | - | PhosphatidylCholine acyl-alkyl C38:2 / PhosphatidylCholine acyl-alkyl C40:0 | PC ae C38:2 / PC ae C40:0 | 1.102 | 8.10*10^-07^ | Glycerophospholipid / Glycerophospholipid |
| ND5 | 12825 | - | T > C | - | lysoPhosphatidylCholine acyl C14:0 / PhosphatidylCholine diacyl C38:5 | lysoPC a C14:0 / PC aa C38:5 | 0.762 | 8.95*10^-07^ | Glycerophospholipid / Glycerophospholipid |
| CYB | 15373 | rs1556424578 | A > G | synonymous | PhosphatidylCholine diacyl C40:5 / PhosphatidylCholine diacyl C40:4 | PC aa C40:5 / PC aa C40:4 | 2.546 | 9.36*10^-07^ | Glycerophospholipid / Glycerophospholipid |
| ND1 | 3392 | - | G > A | - | PhosphatidylCholine diacyl C40:3 / Shingomyeline C 16:0 | PC aa C40:3 / SM C16:0 | 1.075 | 9.72*10^-07^ | Glycerophospholipid / Sphingolipid |
| ND4 | 11493 | - | G > A | - | PhosphatidylCholine diacyl C30:0 / PhosphatidylCholine acyl-alkyl C38:4 | PC aa C30:0 / PC ae C38:4 | 0.754 | 9.78*10^-07^ | Glycerophospholipid / Glycerophospholipid |
| ND4L | 10689 | rs879102108 | G > A | missense | PhosphatidylCholine acyl-alkyl C40:3 / PhosphatidylCholine diacyl C36:6 | PC ae C40:3 / PC aa C36:6 | 0.649 | 9.92*10^-07^ | Glycerophospholipid / Glycerophospholipid |
| ATP6 | 9053 | rs199646902 | G > A | missense | Hydroxyshingomyeline C 16:1 / Shingomyeline C 20:2 | SM (OH) C16:1 / SM C20:2 | 1.779 | 1.03*10^-06^ | Sphingolipid / Sphingolipid |
| ND4L | 10689 | rs879102108 | G > A | missense | Hydroxyshingomyeline C 22:2 / PhosphatidylCholine diacyl C36:6 | SM (OH) C22:2 / PC aa C36:6 | 0.638 | 1.03*10^-06^ | Sphingolipid / Glycerophospholipid |
| tRNA | 3271 | rs199474658 | T > C | - | lysoPhosphatidylCholine acyl C14:0 / PhosphatidylCholine diacyl C30:0 | lysoPC a C14:0 / PC aa C30:0 | 0.9 | 1.06*10^-06^ | Glycerophospholipid / Glycerophospholipid |
| tRNA | 4348 | - | A > G | - | lysoPhosphatidylCholine acyl C28:1 / PhosphatidylCholine acyl-alkyl C42:0 | lysoPC a C28:1 / PC ae C42:0 | 0.86 | 1.09*10^-06^ | Glycerophospholipid / Glycerophospholipid |
| ND5 | 14141 | - | T > C | - | PhosphatidylCholine acyl-alkyl C40:5 / Hexenoyl-L-carnitine | PC ae C40:5 / C6:1 | 0.924 | 1.09*10^-06^ | Glycerophospholipid / Acylcarnitine |
| ND5 | 14141 | - | T > C | - | PhosphatidylCholine acyl-alkyl C40:4 / Hexenoyl-L-carnitine | PC ae C40:4 / C6:1 | 0.894 | 1.09*10-06 | Glycerophospholipid / Acylcarnitine |
| CO3 | 9441 | - | C > T | - | PhosphatidylCholine acyl-alkyl C40:4 / PhosphatidylCholine diacyl C32:0 | PC ae C40:4 / PC aa C32:0 | 0.939 | 1.13*10^-06^ | Glycerophospholipid / Glycerophospholipid |
| ND5 | 14053 | rs200134839 | A > G | missense | PhosphatidylCholine diacyl C36:1 / PhosphatidylCholine diacyl C38:3 | PC aa C36:1 / PC aa C38:3 | 1.599 | 1.17*10^-06^ | Glycerophospholipid / Glycerophospholipid |
| ND4 | 11649 | rs1603223370 | T > C | missense | PhosphatidylCholine acyl-alkyl C40:2 / PhosphatidylCholine acyl-alkyl C38:0 | PC ae C40:2 / PC ae C38:0 | 0.968 | 1.21*10^-06^ | Glycerophospholipid / Glycerophospholipid |
| HVR II | 64 | rs3883917 | C > T | - | PhosphatidylCholine acyl-alkyl C36:0 / PhosphatidylCholine acyl-alkyl C38:1 | PC ae C36:0 / PC ae C38:1 | 1.729 | 1.23*10^-06^ | Glycerophospholipid / Glycerophospholipid |
| 12SrRNA | 715 | - | G > A | - | Acetyl-L-carnitine / Octanoyl-L-carnitine | C2 / C8 | 0.721 | 1.23*10^-06^ | Acylcarnitine / Acylcarnitine |
| 12SrRNA | 715 | - | G > A | - | Acetyl-L-carnitine / Decanoyl-L-carnitine | C2 / C10 | 0.677 | 1.25*10^-06^ | Acylcarnitine / Acylcarnitine |
| ND5 | 14141 | - | T > C | - | PhosphatidylCholine acyl-alkyl C42:5 / Hexenoyl-L-carnitine | PC ae C42:5 / C6:1 | 0.838 | 1.26*10^-06^ | Glycerophospholipid / Acylcarnitine |
| tRNA | 4348 | - | A > G | - | Hydroxyshingomyeline C 14:1 / PhosphatidylCholine acyl-alkyl C42:0 | SM (OH) C14:1 / PC ae C42:0 | 0.737 | 1.28*10^-06^ | Sphingolipid / Glycerophospholipid |
| ATP6 | 9053 | rs199646902 | G > A | missense | Hydroxyshingomyeline C 22:2 / Shingomyeline C 20:2 | SM (OH) C22:2 / SM C20:2 | 1.714 | 1.32*10^-06^ | Sphingolipid / Sphingolipid |
| ND4 | 11875 | rs1556424006 | T > C | synonymous | Shingomyeline C 18:1 / PhosphatidylCholine acyl-alkyl C36:4 | SM C18:1 / PC ae C36:4 | 0.778 | 1.32*10^-06^ | Sphingolipid / Glycerophospholipid |
| tRNA | 5615 | - | A > G | - | PhosphatidylCholine acyl-alkyl C34:2 / lysoPhosphatidylCholine acyl C20:3 | PC ae C34:2 / lysoPC a C20:3 | 0.679 | 1.32*10^-06^ | Glycerophospholipid / Glycerophospholipid |
| CO3 | 9441 | - | C > T | - | PhosphatidylCholine acyl-alkyl C40:5 / PhosphatidylCholine diacyl C32:0 | PC ae C40:5 / PC aa C32:0 | 0.997 | 1.33*10^-06^ | Glycerophospholipid / Glycerophospholipid |
| ND5 | 12718 | - | A > G | - | Shingomyeline C 24:1 / PhosphatidylCholine diacyl C42:2 | SM C24:1 / PC aa C42:2 | 0.921 | 1.33*10^-06^ | Sphingolipid / Glycerophospholipid |
| CO1 | 6040 | rs1556423072 | A > G | missense | Pimelyl-L-carnitine / Tetradecenoyl-L-carnitine | C7-DC / C14:1 | 1.328 | 1.41*10^-06^ | Acylcarnitine / Acylcarnitine |
| HVR I | 16234 | rs368259300 | C > T | - | Hexose / Histidine | H1 / His | 4.175 | 1.42*10^-06^ | Sugar / Amino Acid |
| ND4L | 10689 | rs879102108 | G > A | missense | PhosphatidylCholine acyl-alkyl C38:2 / PhosphatidylCholine diacyl C36:6 | PC ae C38:2 / PC aa C36:6 | 0.637 | 1.49*10^-06^ | Glycerophospholipid / Glycerophospholipid |
| ND5 | 14053 | rs200134839 | A > G | missense | lysoPhosphatidylCholine acyl C18:1 / lysoPhosphatidylCholine acyl C20:3 | lysoPC a C18:1 / lysoPC a C20:3 | 1.336 | 1.49*10^-06^ | Glycerophospholipid / Glycerophospholipid |
| ND5 | 14139 | rs878918283 | A > G | synonymous | PhosphatidylCholine diacyl C42:4 / Shingomyeline C 16:0 | PC aa C42:4 / SM C16:0 | 3.723 | 1.50*10^-06^ | Glycerophospholipid / Sphingolipid |
| ND1 | 4175 | - | G > A | - | Shingomyeline C 26:0 / Octadecadienyl-L-carnitine | SM C26:0 / C18:2 | 0.529 | 1.52*10^-06^ | Sphingolipid / Acylcarnitine |
| ATP6 | 8715 | rs1556423525 | T > C | synonymous | PhosphatidylCholine acyl-alkyl C42:1 / PhosphatidylCholine acyl-alkyl C38:3 | PC ae C42:1 / PC ae C38:3 | 2.042 | 1.55*10^-06^ | Glycerophospholipid / Glycerophospholipid |
| CO2 | 8020 | rs879077802 | G > A | synonymous | PhosphatidylCholine acyl-alkyl C40:2 / lysoPhosphatidylCholine acyl C28:0 | PC ae C40:2 / lysoPC a C28:0 | 0.711 | 1.57*10^-06^ | Glycerophospholipid / Glycerophospholipid |
| HVR II | 206 | - | T > C | - | PhosphatidylCholine acyl-alkyl C36:0 / Hydroxytetradecenoyl-L-carnitine | PC ae C36:0 / C14:1-OH | 0.712 | 1.58*10^-06^ | Glycerophospholipid / Acylcarnitine |
| 16SrRNA | 1782 | - | G > A | - | PhosphatidylCholine acyl-alkyl C34:0 / PhosphatidylCholine diacyl C34:3 | PC ae C34:0 / PC aa C34:3 | 0.969 | 1.60*10^-06^ | Glycerophospholipid / Glycerophospholipid |
| CO1 | 6207 | - | T > C | - | Shingomyeline C 18:0 / Glutaconyl-L-carnitine | SM C18:0 / C5:1-DC | 0.729 | 1.63*10^-06^ | Sphingolipid / Glycerophospholipid |
| tRNA | 3243 | rs199474657 | A > G | - | PhosphatidylCholine diacyl C30:0 / Proline | PC aa C30:0 / Pro | 0.622 | 1.63*10^-06^ | Glycerophospholipid / Amino Acid |
| CO1 | 6994 | - | A > G | - | PhosphatidylCholine acyl-alkyl C40:2 / lysoPhosphatidylCholine acyl C16:1 | PC ae C40:2 / lysoPC a C16:1 | 0.484 | 1.64*10^-06^ | Glycerophospholipid / Glycerophospholipid |
| CO1 | 6484 | - | T > C | - | PhosphatidylCholine diacyl C34:1 / lysoPhosphatidylCholine acyl C24:0 | PC aa C34:1 / lysoPC a C24:0 | 0.781 | 1.67*10^-06^ | Glycerophospholipid / Glycerophospholipid |
| 12SrRNA | 1422 | - | G > A | - | PhosphatidylCholine acyl-alkyl C34:0 / lysoPhosphatidylCholine acyl C28:1 | PC ae C34:0 / lysoPC a C28:1 | 0.987 | 1.72*10^-06^ | Glycerophospholipid / Glycerophospholipid |
| CO3 | 9441 | - | C > T | - | PhosphatidylCholine acyl-alkyl C40:4 / PhosphatidylCholine diacyl C30:0 | PC ae C40:4 / PC aa C30:0 | 0.635 | 1.73*10^-06^ | Glycerophospholipid / Glycerophospholipid |
| CYB | 15821 | - | T > C | - | PhosphatidylCholine diacyl C36:4 / Shingomyeline C 24:0 | PC aa C36:4 / SM C24:0 | 0.841 | 1.76*10^-06^ | Glycerophospholipid / Sphingolipid |
| CO2 | 7809 | - | T > C | - | Hydroxyshingomyeline C 16:1 / Shingomyeline C 18:1 | SM (OH) C16:1 / SM C18:1 | 1.134 | 1.83*10^-06^ | Sphingolipid / Sphingolipid |
| ATP6 | 9045 | - | A > G | - | Dodecanoyl-L-carnitine / Malonyl-L-carnitine , Hydroxybutyryl-L-carnitine | C12 / C4-OH (C3-DC) | 0.343 | 1.85*10^-06^ | Acylcarnitine / Acylcarnitine |
| HVR II | 223 | - | T > C | - | PhosphatidylCholine acyl-alkyl C40:3 / Serine | PC ae C40:3 / Ser | 0.958 | 1.91*10^-06^ | Glycerophospholipid / Amino Acid |
| ATP8 | 8389 | rs386829032 | A > G | synonymous | lysoPhosphatidylCholine acyl C18:1 / PhosphatidylCholine diacyl C36:4 | lysoPC a C18:1 / PC aa C36:4 | 0.667 | 1.92*10^-06^ | Glycerophospholipid / Glycerophospholipid |
| HVR I | 16281 | - | A > G | - | Tetradecenoyl-L-carnitine / Tetradecanoyl-L-carnitine | C14:1 / C14 | 1.547 | 1.97*10^-06^ | Acylcarnitine / Acylcarnitine |
| CO1 | 7115 | - | C > T | - | PhosphatidylCholine acyl-alkyl C40:3 / PhosphatidylCholine acyl-alkyl C40:0 | PC ae C40:3 / PC ae C40:0 | 1.16 | 1.98*10^-06^ | Glycerophospholipid / Glycerophospholipid |
| tRNA | 12323 | - | T > C | - | PhosphatidylCholine acyl-alkyl C34:3 / PhosphatidylCholine acyl-alkyl C36:3 | PC ae C34:3 / PC ae C36:3 | 1.041 | 2.04*10^-06^ | Glycerophospholipid / Glycerophospholipid |
| 12SrRNA | 856 | rs1603218502 | A > G | - | Hydroxyshingomyeline C 22:1 / lysoPhosphatidylCholine acyl C28:1 | SM (OH) C22:1 / lysoPC a C28:1 | 0.848 | 2.05*10^-06^ | Sphingolipid / Glycerophospholipid |
| tRNA | 15915 | rs1603225588 | G > A | - | lysoPhosphatidylCholine acyl C16:0 / Shingomyeline C 26:1 | lysoPC a C16:0 / SM C26:1 | 0.64 | 2.06*10^-06^ | Glycerophospholipid / Sphingolipid |
| ND5 | 14141 | - | T > C | - | Hydroxyshingomyeline C 22:2 / Hexenoyl-L-carnitine | SM (OH) C22:2 / C6:1 | 0.82 | 2.07*10^-06^ | Sphingolipid / Acylcarnitine |
| ATP8 | 8389 | rs386829032 | A > G | synonymous | lysoPhosphatidylCholine acyl C18:2 / PhosphatidylCholine acyl-alkyl C38:4 | lysoPC a C18:2 / PC ae C38:4 | 0.592 | 2.13*10^-06^ | Glycerophospholipid / Glycerophospholipid |
| 12SrRNA | 873 | - | G > A | - | PhosphatidylCholine acyl-alkyl C34:2 / PhosphatidylCholine diacyl C40:1 | PC ae C34:2 / PC aa C40:1 | 0.867 | 2.16*10^-06^ | Glycerophospholipid / Glycerophospholipid |
| CYB | 15821 | - | T > C | - | PhosphatidylCholine acyl-alkyl C40:1 / PhosphatidylCholine diacyl C42:2 | PC ae C40:1 / PC aa C42:2 | 0.884 | 2.16*10^-06^ | Glycerophospholipid / Glycerophospholipid |
| tRNA | 12161 | rs386829147 | T > C | - | Methylmalonyl-L-carnitine , Hydroxyvaleryl-L-carnitine / Shingomyeline C 26:0 | C5-OH (C3-DC-M) / SM C26:0 | 0.894 | 2.19*10^-06^ | Acylcarnitine / Sphingolipid |
| tRNA | 12161 | rs386829147 | T > C | - | Methylmalonyl-L-carnitine , Hydroxyvaleryl-L-carnitine / PhosphatidylCholine acyl-alkyl C40:2 | C5-OH (C3-DC-M) / PC ae C40:2 | 1.02 | 2.20*10^-06^ | Acylcarnitine / Glycerophospholipid |
| CO1 | 6994 | - | A > G | - | PhosphatidylCholine acyl-alkyl C32:2 / lysoPhosphatidylCholine acyl C16:1 | PC ae C32:2 / lysoPC a C16:1 | 0.561 | 2.22*10^-06^ | Glycerophospholipid / Glycerophospholipid |
| ATP6 | 9028 | rs1603221990 | C > T | missense | PhosphatidylCholine acyl-alkyl C40:4 / PhosphatidylCholine diacyl C36:3 | PC ae C40:4 / PC aa C36:3 | 0.868 | 2.28*10^-06^ | Glycerophospholipid / Glycerophospholipid |
| CO2 | 7809 | - | T > C | - | Hydroxyshingomyeline C 16:1 / Shingomyeline C 16:1 | SM (OH) C16:1 / SM C16:1 | 1.057 | 2.29*10^-06^ | Sphingolipid / Sphingolipid |
| CO1 | 6994 | - | A > G | - | PhosphatidylCholine diacyl C32:3 / lysoPhosphatidylCholine acyl C16:1 | PC aa C32:3 / lysoPC a C16:1 | 0.549 | 2.31*10^-06^ | Glycerophospholipid / Glycerophospholipid |
| ND1 | 3360 | rs879023598 | A > G | synonymous | Hydroxyshingomyeline C 16:1 / Hydroxyshingomyeline C 24:1 | SM (OH) C16:1 / SM (OH) C24:1 | 1.04 | 2.35*10^-06^ | Sphingolipid / Sphingolipid |
| ND4L | 10689 | rs879102108 | G > A | missense | PhosphatidylCholine acyl-alkyl C32:2 / PhosphatidylCholine diacyl C36:6 | PC ae C32:2 / PC aa C36:6 | 0.66 | 2.36*10^-06^ | Glycerophospholipid / Glycerophospholipid |
| ND5 | 12977 | - | T > C | - | lysoPhosphatidylCholine acyl C17:0 / PhosphatidylCholine acyl-alkyl C38:1 | lysoPC a C17:0 / PC ae C38:1 | 0.605 | 2.37*10^-06^ | Glycerophospholipid / Glycerophospholipid |
| ND4 | 11342 | - | A > G | - | PhosphatidylCholine acyl-alkyl C40:2 / Hexadecanoyl-L-carnitine | PC ae C40:2 / C16 | 0.777 | 2.43*10^-06^ | Glycerophospholipid / Acylcarnitine |
| ATP6 | 9045 | - | A > G | - | Dodecenoyl-L-carnitine / Malonyl-L-carnitine , Hydroxybutyryl-L-carnitine | C12:1 / C4-OH (C3-DC) | 0.376 | 2.43*10^-06^ | Acylcarnitine / Acylcarnitine |
| ND5 | 12666 | rs1603223855 | A > G | synonymous | DL-Carnitine / Methylglutaryl-L-carnitine | C0 / C5-M-DC | 0.729 | 2.44*10^-06^ | Acylcarnitine / Acylcarnitine |
| ND4 | 11342 | - | A > G | - | PhosphatidylCholine acyl-alkyl C40:2 / Hydroxyhexadecenoyl-L-carnitine | PC ae C40:2 / C16:1-OH | 0.848 | 2.46*10^-06^ | Glycerophospholipid / Acylcarnitine |
| HVR II | 249 | rs1556422405 | A > G | - | PhosphatidylCholine diacyl C24:0 / PhosphatidylCholine diacyl C26:0 | PC aa C24:0 / PC aa C26:0 | 1.855 | 2.51*10^-06^ | Glycerophospholipid / Glycerophospholipid |
| tRNA | 7503 | - | C > T | - | lysoPhosphatidylCholine acyl C14:0 / lysoPhosphatidylCholine acyl C20:4 | lysoPC a C14:0 / lysoPC a C20:4 | 0.522 | 2.51*10^-06^ | Glycerophospholipid / Glycerophospholipid |
| 12SrRNA | 715 | - | G > A | - | Fumaryl-L-carnitine , Hexanoyl-L-carnitine / Decenoyl-L-carnitine | C6 (C4:1-DC) / C10:1 | 1.022 | 2.53*10^-06^ | Acylcarnitine / Acylcarnitine |
| ND5 | 12825 | - | T > C | - | lysoPhosphatidylCholine acyl C14:0 / PhosphatidylCholine diacyl C42:6 | lysoPC a C14:0 / PC aa C42:6 | 0.791 | 2.53*10^-06^ | Glycerophospholipid / Glycerophospholipid |
| ND5 | 13565 | rs56039545 | C > T | missense | PhosphatidylCholine diacyl C42:2 / PhosphatidylCholine acyl-alkyl C42:1 | PC aa C42:2 / PC ae C42:1 | 1.12 | 2.53*10^-06^ | Glycerophospholipid / Glycerophospholipid |
| ND6 | 14548 | rs1556424457 | A > G | synonymous | PhosphatidylCholine acyl-alkyl C38:1 / PhosphatidylCholine diacyl C42:2 | PC ae C38:1 / PC aa C42:2 | 0.569 | 2.54*10^-06^ | Glycerophospholipid / Glycerophospholipid |
| ND4 | 10962 | - | T > C | - | PhosphatidylCholine acyl-alkyl C34:1 / PhosphatidylCholine diacyl C40:2 | PC ae C34:1 / PC aa C40:2 | 0.847 | 2.61*10^-06^ | Glycerophospholipid / Glycerophospholipid |
| ND4 | 11649 | rs1603223370 | T > C | missense | PhosphatidylCholine acyl-alkyl C40:2 / PhosphatidylCholine diacyl C36:6 | PC ae C40:2 / PC aa C36:6 | 0.694 | 2.64*10^-06^ | Glycerophospholipid / Glycerophospholipid |
| HVR I | 16299 | rs879021682 | A > G | - | lysoPhosphatidylCholine acyl C28:0 / PhosphatidylCholine diacyl C42:5 | lysoPC a C28:0 / PC aa C42:5 | 1.312 | 2.65*10^-06^ | Glycerophospholipid / Glycerophospholipid |
| ND6 | 14623 | - | C > T | - | Shingomyeline C 24:1 / PhosphatidylCholine acyl-alkyl C34:3 | SM C24:1 / PC ae C34:3 | 0.993 | 2.66*10^-06^ | Sphingolipid / Glycerophospholipid |
| CO1 | 5972 | rs1603220208 | C > T | synonymous | Glutaconyl-L-carnitine / Fumaryl-L-carnitine , Hexanoyl-L-carnitine | C5:1:DC / C6 (C4:1-DC) | 1.244 | 2.67*10^-06^ | Glycerophospholipid / Acylcarnitine |
| 12SrRNA | 856 | rs1603218502 | A > G | - | Hydroxyshingomyeline C 14:1 / lysoPhosphatidylCholine acyl C28:1 | SM (OH) C14:1 / lysoPC a C28:1 | 0.895 | 2.72*10^-06^ | Sphingolipid / Glycerophospholipid |
| ND4L | 10689 | rs879102108 | G > A | missense | PhosphatidylCholine acyl-alkyl C40:2 / PhosphatidylCholine diacyl C36:6 | PC ae C40:2 / PC aa C36:6 | 0.665 | 2.77*10^-06^ | Glycerophospholipid / Glycerophospholipid |
| ND4 | 11649 | rs1603223370 | T > C | missense | PhosphatidylCholine acyl-alkyl C40:2 / PhosphatidylCholine acyl-alkyl C32:1 | PC ae C40:2 / PC ae C32:1 | 1.091 | 2.82*10^-06^ | Glycerophospholipid / Glycerophospholipid |
| ND4 | 11069 | rs1603223091 | A > G | missense | PhosphatidylCholine acyl-alkyl C36:0 / Shingomyeline C 26:0 | PC ae C36:0 / SM C26:0 | 0.823 | 2.84*10^-06^ | Glycerophospholipid / Sphingolipid |
| ND4 | 11902 | rs1603223459 | G > A | synonymous | PhosphatidylCholine acyl-alkyl C36:2 / PhosphatidylCholine diacyl C38:0 | PC ae C36:2 / PC aa C38:0 | 0.769 | 2.86*10^-06^ | Glycerophospholipid / Glycerophospholipid |
| ND4L | 10645 | - | T > C | - | Shingomyeline C 26:0 / PhosphatidylCholine diacyl C36:0 | SM C26:0 / PC aa C36:0 | 0.621 | 2.88*10^-06^ | Sphingolipid / Glycerophospholipid |
| ND4 | 11017 | rs878902408 | T > C | synonymous | PhosphatidylCholine acyl-alkyl C32:2 / PhosphatidylCholine diacyl C40:1 | PC ae C32:2 / PC aa C40:1 | 1.036 | 2.89*10^-06^ | Glycerophospholipid / Glycerophospholipid |
| CO1 | 6510 | rs1603220518 | G > A | missense | PhosphatidylCholine diacyl C40:3 / Octadecanoyl-L-carnitine | PC aa C40:3 / C18 | 0.677 | 2.96*10^-06^ | Glycerophospholipid / Acylcarnitine |
| tRNA | 5615 | - | A > G | - | PhosphatidylCholine acyl-alkyl C36:3 / lysoPhosphatidylCholine acyl C20:3 | PC ae C36:3 / lysoPC a C20:3 | 0.735 | 2.97*10^-06^ | Glycerophospholipid / Glycerophospholipid |
| ND4L | 10645 | - | T > C | - | Shingomyeline C 26:0 / PhosphatidylCholine acyl-alkyl C38:0 | SM C26:0 / PC ae C38:0 | 0.591 | 2.98*10^-06^ | Sphingolipid / Glycerophospholipid |
| tRNA | 4348 | - | A > G | - | Hydroxyshingomyeline C 16:1 / PhosphatidylCholine diacyl C40:6 | SM (OH) C16:1 / PC aa C40:6 | 0.559 | 3.03*10^-06^ | Sphingolipid / Glycerophospholipid |
| HVR I | 16345 | - | A > G | - | PhosphatidylCholine acyl-alkyl C38:3 / PhosphatidylCholine diacyl C36:2 | PC ae C38:3 / PC aa C36:2 | 1.247 | 3.03*10^-06^ | Glycerophospholipid / Glycerophospholipid |
| CO3 | 9905 | rs369730420 | T > C | synonymous | PhosphatidylCholine acyl-alkyl C30:2 / PhosphatidylCholine acyl-alkyl C42:1 | PC ae C30:2 / PC ae C42:1 | 0.904 | 3.04*10^-06^ | Glycerophospholipid / Glycerophospholipid |
| ND5 | 13356 | rs1603224159 | T > C | synonymous | PhosphatidylCholine diacyl C32:1 / Shingomyeline C 24:0 | PC aa C32:1 / SM C24:0 | 0.689 | 3.07*10^-06^ | Glycerophospholipid / Sphingolipid |
| CO3 | 9490 | rs1603222345 | C > T | missense | Shingomyeline C 16:1 / PhosphatidylCholine diacyl C40:1 | SM C16:1 / PC aa C40:1 | 0.939 | 3.13*10^-06^ | Sphingolipid / Glycerophospholipid |
| CYB | 15141 | rs1603225108 | T > C | missense | lysoPhosphatidylCholine acyl C17:0 / lysoPhosphatidylCholine acyl C28:1 | lysoPC a C17:0 / lysoPC a C28:1 | 0.532 | 3.16*10^-06^ | Glycerophospholipid / Glycerophospholipid |
| ND4L | 10689 | rs879102108 | G > A | missense | PhosphatidylCholine acyl-alkyl C30:2 / PhosphatidylCholine diacyl C36:6 | PC ae C30:2 / PC aa C36:6 | 0.636 | 3.18*10^-06^ | Glycerophospholipid / Glycerophospholipid |
| CO3 | 9441 | - | C > T | - | PhosphatidylCholine acyl-alkyl C40:5 / PhosphatidylCholine diacyl C30:0 | PC ae C40:5 / PC aa C30:0 | 0.634 | 3.19*10^-06^ | Glycerophospholipid / Glycerophospholipid |
| CYB | 15481 | rs1603225305 | C > T | synonymous | lysoPhosphatidylCholine acyl C28:1 / PhosphatidylCholine acyl-alkyl C38:1 | lysoPC a C28:1 / PC ae C38:1 | 1.015 | 3.21*10^-06^ | Glycerophospholipid / Glycerophospholipid |
| CO1 | 6687 | - | T > C | - | PhosphatidylCholine diacyl C36:1 / Hydroxyhexadecadienyl-L-carnitine | PC aa C36:1 / C16:2-OH | 0.801 | 3.22*10^-06^ | Glycerophospholipid / Acylcarnitine |
| ND4 | 11017 | rs878902408 | T > C | synonymous | PhosphatidylCholine acyl-alkyl C32:1 / PhosphatidylCholine diacyl C40:1 | PC ae C32:1 / PC aa C40:1 | 0.975 | 3.28*10^-06^ | Glycerophospholipid / Glycerophospholipid |
| ND1 | 3520 | rs1603218996 | A > G | missense | PhosphatidylCholine acyl-alkyl C40:5 / Shingomyeline C 24:0 | PC ae C40:5 / SM C24:0 | 0.896 | 3.28*10^-06^ | Glycerophospholipid / Sphingolipid |
| ND5 | 12994 | rs1603223993 | G > A | missense | PhosphatidylCholine acyl-alkyl C40:1 / PhosphatidylCholine diacyl C36:6 | PC ae C40:1 / PC aa C36:6 | 0.722 | 3.30*10^-06^ | Glycerophospholipid / Glycerophospholipid |
| ND4 | 11875 | rs1556424006 | T > C | synonymous | Shingomyeline C 18:1 / PhosphatidylCholine acyl-alkyl C38:5 | SM C18:1 / PC ae C38:5 | 0.838 | 3.31*10^-06^ | Sphingolipid / Glycerophospholipid |
| ND4 | 10962 | - | T > C | - | PhosphatidylCholine acyl-alkyl C34:1 / PhosphatidylCholine diacyl C42:4 | PC ae C34:1 / PC aa C42:4 | 0.957 | 3.31*10^-06^ | Glycerophospholipid / Glycerophospholipid |
| ND4L | 10632 | rs878888873 | T > C | synonymous | PhosphatidylCholine acyl-alkyl C36:0 / PhosphatidylCholine diacyl C40:3 | PC ae C36:0 / PC aa C40:3 | 1.604 | 3.39*10^-06^ | Glycerophospholipid / Glycerophospholipid |
| ND3 | 10217 | rs1556423786 | A > G | synonymous | Tetradecanoyl-L-carnitine / Hydroxytetradecadienyl-L-carnitine | C14 / C14:2-OH | 1.285 | 3.40*10^-06^ | Acylcarnitine / Acylcarnitine |
| ATP6 | 8643 | - | C > T | - | Shingomyeline C 24:1 / PhosphatidylCholine acyl-alkyl C38:0 | SM C24:1 / PC ae C38:0 | 0.589 | 3.46*10^-06^ | Sphingolipid / Glycerophospholipid |
| ND5 | 12488 | - | C > T | - | PhosphatidylCholine acyl-alkyl C40:2 / Hydroxyhexadecadienyl-L-carnitine | PC ae C40:2 / C16:2-OH | 0.625 | 3.46*10^-06^ | Glycerophospholipid / Acylcarnitine |
| CYB | 15799 | rs1603225506 | A > G | synonymous | Methylmalonyl-L-carnitine , Hydroxyvaleryl-L-carnitine / Decadienyl-L-carnitine | C5-OH (C3-DC-M)/ C10:2 | 0.672 | 3.47*10^-06^ | Acylcarnitine / Acylcarnitine |
| ATP8 | 8389 | rs386829032 | A > G | synonymous | lysoPhosphatidylCholine acyl C18:2 / PhosphatidylCholine acyl-alkyl C36:5 | lysoPC a C18:2 / PC ae C36:5 | 0.549 | 3.49*10^-06^ | Glycerophospholipid / Glycerophospholipid |
| ATP8 | 8389 | rs386829032 | A > G | synonymous | lysoPhosphatidylCholine acyl C18:1 / Hexadecanoyl-L-carnitine | lysoPC a C18:1 / C16 | 0.644 | 3.51*10^-06^ | Glycerophospholipid / Acylcarnitine |
| ND4 | 10962 | - | T > C | - | PhosphatidylCholine acyl-alkyl C34:1 / PhosphatidylCholine diacyl C42:5 | PC ae C34:1 / PC aa C42:5 | 0.795 | 3.54*10^-06^ | Glycerophospholipid / Glycerophospholipid |
| ATP6 | 8574 | rs1603221595 | C > T | synonymous | Malonyl-L-carnitine , Hydroxybutyryl-L-carnitine / Dodecenoyl-L-carnitine | C4-OH (C3-DC) / C12:1 | 0.676 | 3.58*10^-06^ | Acylcarnitine / Acylcarnitine |
| ND4 | 10962 | - | T > C | - | PhosphatidylCholine acyl-alkyl C34:1 / PhosphatidylCholine diacyl C40:3 | PC ae C34:1 / PC aa C40:3 | 1.016 | 3.60*10^-06^ | Glycerophospholipid / Glycerophospholipid |
| CYB | 15746 | rs386829260 | A > G | missense | Proline / Threonine | Pro / Thr | 0.748 | 3.64*10^-06^ | Amino Acid / Amino Acid |
| CO1 | 6510 | rs1603220518 | G > A | missense | PhosphatidylCholine diacyl C36:1 / Octadecanoyl-L-carnitine | PC aa C36:1 / C18 | 0.736 | 3.65*10^-06^ | Glycerophospholipid / Acylcarnitine |
| ND4L | 10645 | - | T > C | - | Shingomyeline C 26:0 / PhosphatidylCholine diacyl C36:4 | SM C26:0 / PC aa C36:4 | 0.588 | 3.70*10^-06^ | Sphingolipid / Glycerophospholipid |
| CYB | 15042 | rs1603225039 | G > A | missense | Nonayl-L-carnitine / PhosphatidylCholine diacyl C36:2 | C9 / PC aa C36:2 | 0.564 | 3.72*10^-06^ | Acylcarnitine / Glycerophospholipid |
| ND5 | 14141 | - | T > C | - | Hydroxyshingomyeline C 14:1 / Hexenoyl-L-carnitine | SM (OH) C14:1 / C6:1 | 0.749 | 3.72*10^-06^ | Sphingolipid / Acylcarnitine |
| ND2 | 5075 | rs1603219767 | T > C | synonymous | Hydroxyshingomyeline C 22:1 / Hydroxyshingomyeline C 22:2 | SM (OH) C22:1 / SM (OH) C22:2 | 1.884 | 3.73*10^-06^ | Sphingolipid / Sphingolipid |
| ND4 | 11493 | - | G > A | - | PhosphatidylCholine diacyl C30:0 / PhosphatidylCholine acyl-alkyl C36:4 | PC aa C30:0 / PC ae C36:4 | 0.653 | 3.74*10^-06^ | Glycerophospholipid / Glycerophospholipid |
| HVR I | 16299 | rs879021682 | A > G | - | PhosphatidylCholine diacyl C26:0 / PhosphatidylCholine diacyl C42:5 | PC aa C26:0 / PC aa C42:5 | 1.39 | 3.75*10^-06^ | Glycerophospholipid / Glycerophospholipid |
| ND2 | 5348 | rs878922126 | C > T | synonymous | Octadecanoyl-L-carnitine / Hydroxytetradecadienyl-L-carnitine | C18 / C14:2-OH | 1.089 | 3.77*10^-06^ | Acylcarnitine / Acylcarnitine |
| ND5 | 14141 | - | T > C | - | Shingomyeline C 16:0 / Hexenoyl-L-carnitine | SM C16:0 / C6:1 | 0.957 | 3.79*10^-06^ | Sphingolipid / Acylcarnitine |
| ND4 | 11513 | - | C > T | - | PhosphatidylCholine acyl-alkyl C36:1 / Shingomyeline C 24:0 | PC ae C36:1 / SM C24:0 | 0.925 | 3.80*10^-06^ | Glycerophospholipid / Sphingolipid |
| ND1 | 3520 | rs1603218996 | A > G | missense | PhosphatidylCholine acyl-alkyl C42:5 / Shingomyeline C 24:0 | PC ae C42:5 / SM C24:0 | 0.774 | 3.81*10^-06^ | Glycerophospholipid / Sphingolipid |
| ATP8 | 8389 | rs386829032 | A > G | synonymous | lysoPhosphatidylCholine acyl C18:1 / PhosphatidylCholine acyl-alkyl C38:4 | lysoPC a C18:1 / PC ae C38:4 | 0.685 | 3.86*10^-06^ | Glycerophospholipid / Glycerophospholipid |
| ATP8 | 8389 | rs386829032 | A > G | synonymous | lysoPhosphatidylCholine acyl C18:2 / PhosphatidylCholine diacyl C36:3 | lysoPC a C18:2 / PC aa C36:3 | 0.563 | 3.87*10^-06^ | Glycerophospholipid / Glycerophospholipid |
| ND4 | 11493 | - | G > A | - | PhosphatidylCholine diacyl C42:6 / PhosphatidylCholine acyl-alkyl C38:4 | PC aa C42:6 / PC ae C38:4 | 0.929 | 3.95*10^-06^ | Glycerophospholipid / Glycerophospholipid |
| ATP8 | 8389 | rs386829032 | A > G | synonymous | lysoPhosphatidylCholine acyl C18:1 / PhosphatidylCholine diacyl C36:3 | lysoPC a C18:1 / PC aa C36:3 | 0.685 | 4.02*10^-06^ | Glycerophospholipid / Glycerophospholipid |
| 16SrRNA | 1957 | - | A > G | - | PhosphatidylCholine acyl-alkyl C44:6 / PhosphatidylCholine acyl-alkyl C36:4 | PC ae C44:6 / PC ae C36:4 | 0.693 | 4.05*10^-06^ | Glycerophospholipid / Glycerophospholipid |
| tRNA | 618 | rs1603218460 | T > C | - | Hydroxyshingomyeline C 22:1 / Shingomyeline C 16:1 | SM (OH) C22:1 / SM C16:1 | 1.216 | 4.09*10^-06^ | Sphingolipid / Sphingolipid |
| 12SrRNA | 1205 | - | T > C | - | lysoPhosphatidylCholine acyl C16:0 / PhosphatidylCholine acyl-alkyl C34:1 | lysoPC a C16:0 / PC ae C34:1 | 0.732 | 4.14*10^-06^ | Glycerophospholipid / Glycerophospholipid |
| ND5 | 14141 | - | T > C | - | PhosphatidylCholine acyl-alkyl C38:4 / Hexenoyl-L-carnitine | PC ae C38:4 / C6:1 | 0.848 | 4.14*10^-06^ | Glycerophospholipid / Acylcarnitine |
| CO2 | 7589 | - | G > A | - | PhosphatidylCholine acyl-alkyl C40:2 / PhosphatidylCholine acyl-alkyl C38:2 | PC ae C40:2 / PC ae C38:2 | 1.032 | 4.17*10^-06^ | Glycerophospholipid / Glycerophospholipid |
| ND5 | 12825 | - | T > C | - | lysoPhosphatidylCholine acyl C14:0 / PhosphatidylCholine diacyl C34:4 | lysoPC a C14:0 / PC aa C34:4 | 0.683 | 4.25*10^-06^ | Glycerophospholipid / Glycerophospholipid |
| CO3 | 9441 | - | C > T | - | PhosphatidylCholine acyl-alkyl C38:3 / PhosphatidylCholine diacyl C30:0 | PC ae C38:3 / PC aa C30:0 | 0.672 | 4.26*10^-06^ | Glycerophospholipid / Glycerophospholipid |
| 12SrRNA | 715 | - | G > A | - | Fumaryl-L-carnitine , Hexanoyl-L-carnitine / Octanoyl-L-carnitine | C6 (C4:1-DC) / C8 | 1.198 | 4.29*10^-06^ | Acylcarnitine / Acylcarnitine |
| ATP6 | 8643 | - | C > T | - | PhosphatidylCholine acyl-alkyl C44:5 / PhosphatidylCholine acyl-alkyl C38:0 | PC ae C44:5 / PC ae C38:0 | 0.476 | 4.29*10^-06^ | Glycerophospholipid / Glycerophospholipid |
| ND5 | 12825 | - | T > C | - | lysoPhosphatidylCholine acyl C14:0 / PhosphatidylCholine diacyl C36:5 | lysoPC a C14:0 / PC aa C36:5 | 0.488 | 4.34*10^-06^ | Glycerophospholipid / Glycerophospholipid |
| CO2 | 8262 | - | C > T | - | Malonyl-L-carnitine , Hydroxybutyryl-L-carnitine / Glutaconyl-L-carnitine | C4-OH (C3-DC) / C5:1-DC | 0.508 | 4.49*10^-06^ | Acylcarnitine / Glycerophospholipid |
| ATP8 | 8389 | rs386829032 | A > G | synonymous | lysoPhosphatidylCholine acyl C18:1 / PhosphatidylCholine diacyl C38:5 | lysoPC a C18:1 / PC aa C38:5 | 0.676 | 4.52*10^-06^ | Glycerophospholipid / Glycerophospholipid |
| CO1 | 6994 | - | A > G | - | PhosphatidylCholine acyl-alkyl C36:1 / lysoPhosphatidylCholine acyl C16:1 | PC ae C36:1 / lysoPC a C16:1 | 0.538 | 4.57*10^-06^ | Glycerophospholipid / Glycerophospholipid |
| ND4 | 11649 | rs1603223370 | T > C | missense | PhosphatidylCholine acyl-alkyl C40:2 / PhosphatidylCholine acyl-alkyl C32:2 | PC ae C40:2 / PC ae C32:2 | 1.199 | 4.60*10^-06^ | Glycerophospholipid / Glycerophospholipid |
| 12SrRNA | 873 | - | G > A | - | PhosphatidylCholine acyl-alkyl C40:3 / PhosphatidylCholine diacyl C40:1 | PC ae C40:3 / PC aa C40:1 | 1.295 | 4.64*10^-06^ | Glycerophospholipid / Glycerophospholipid |
| 16SrRNA | 2186 | - | C > T | - | PhosphatidylCholine acyl-alkyl C40:6 / lysoPhosphatidylCholine acyl C17:0 | PC ae C40:6 / lysoPC a C17:0 | 0.803 | 4.64*10^-06^ | Glycerophospholipid / Glycerophospholipid |
| ND4L | 10689 | rs879102108 | G > A | missense | PhosphatidylCholine acyl-alkyl C44:3 / PhosphatidylCholine diacyl C36:6 | PC ae C44:3 / PC aa C36:6 | 0.619 | 4.65*10^-06^ | Glycerophospholipid / Glycerophospholipid |
| 16SrRNA | 2087 | - | T > C | - | PhosphatidylCholine acyl-alkyl C34:0 / PhosphatidylCholine diacyl C42:2 | PC ae C34:0 / PC aa C42:2 | 0.75 | 4.67*10^-06^ | Glycerophospholipid / Glycerophospholipid |
| ND4 | 11199 | - | C > T | - | lysoPhosphatidylCholine acyl C17:0 / PhosphatidylCholine acyl-alkyl C34:2 | lysoPC a C17:0 / PC ae C34:2 | 0.641 | 4.71*10^-06^ | Glycerophospholipid / Glycerophospholipid |
| CYB | 15355 | rs527236181 | G > A | synonymous | Hydroxyshingomyeline C 22:1 / lysoPhosphatidylCholine acyl C18:0 | SM (OH) C22:1 / lysoPC a C18:0 | 1.311 | 4.72*10^-06^ | Sphingolipid / Glycerophospholipid |
| ND4 | 11875 | rs1556424006 | T > C | synonymous | Shingomyeline C 16:1 / PhosphatidylCholine acyl-alkyl C36:4 | SM C16:1 / PC ae C36:4 | 0.853 | 4.73*10^-06^ | Sphingolipid / Glycerophospholipid |
| ND5 | 12977 | - | T > C | - | Hydroxyshingomyeline C 16:1 / PhosphatidylCholine acyl-alkyl C38:1 | SM (OH) C16:1 / PC ae C38:1 | 0.594 | 4.75*10^-06^ | Sphingolipid / Glycerophospholipid |
| ND4 | 11493 | - | G > A | - | PhosphatidylCholine diacyl C32:0 / PhosphatidylCholine acyl-alkyl C38:4 | PC aa C32:0 / PC ae C38:4 | 1.011 | 4.76*10^-06^ | Glycerophospholipid / Glycerophospholipid |
| ND4 | 11902 | rs1603223459 | G > A | synonymous | PhosphatidylCholine acyl-alkyl C36:2 / PhosphatidylCholine diacyl C36:0 | PC ae C36:2 / PC aa C36:0 | 0.693 | 4.78*10^-06^ | Glycerophospholipid / Glycerophospholipid |
| ND4 | 10962 | - | T > C | - | PhosphatidylCholine acyl-alkyl C36:1 / PhosphatidylCholine diacyl C40:2 | PC ae C36:1 / PC aa C40:2 | 0.837 | 4.87*10^-06^ | Glycerophospholipid / Glycerophospholipid |
| ATP6 | 9070 | rs879190502 | T > C | missense | Hexadecanoyl-L-carnitine / Shingomyeline C 16:1 | C16 / SM C16:1 | 0.764 | 4.90*10^-06^ | Acylcarnitine / Sphingolipid |
| 12SrRNA | 1028 | - | G > A | - | PhosphatidylCholine acyl-alkyl C34:1 / PhosphatidylCholine acyl-alkyl C34:3 | PC ae C34:1 / PC ae C34:3 | 0.708 | 5.00*10^-06^ | Glycerophospholipid / Glycerophospholipid |
| ND5 | 14053 | rs200134839 | A > G | missense | PhosphatidylCholine diacyl C34:1 / PhosphatidylCholine diacyl C38:3 | PC aa C34:1 / PC aa C38:3 | 1.351 | 5.00*10^-06^ | Glycerophospholipid / Glycerophospholipid |
| ATP8 | 8389 | rs386829032 | A > G | synonymous | lysoPhosphatidylCholine acyl C18:1 / PhosphatidylCholine diacyl C34:4 | lysoPC a C18:1 / PC aa C34:4 | 0.507 | 5.00*10^-06^ | Glycerophospholipid / Glycerophospholipid |
| 16SrRNA | 3144 | rs1556422678 | A > G | - | PhosphatidylCholine acyl-alkyl C36:2 / PhosphatidylCholine acyl-alkyl C42:2 | PC ae C36:2 / PC ae C42:2 | 0.972 | 5.02*10^-06^ | Glycerophospholipid / Glycerophospholipid |
| CO1 | 6484 | - | T > C | - | PhosphatidylCholine diacyl C32:0 / lysoPhosphatidylCholine acyl C24:0 | PC aa C32:0 / lysoPC a C24:0 | 0.795 | 5.02*10^-06^ | Glycerophospholipid / Glycerophospholipid |
| HVR I | 16286 | rs1556424846 | C > T | - | PhosphatidylCholine diacyl C34:4 / PhosphatidylCholine diacyl C32:2 | PC aa C34:4 / PC aa C32:2 | 1.221 | 5.03*10^-06^ | Glycerophospholipid / Glycerophospholipid |
| ATP6 | 9070 | rs879190502 | T > C | missense | Octadecenoyl-L-carnitine / Shingomyeline C 16:1 | C18:1 / SM C16:1 | 0.697 | 5.05*10^-06^ | Acylcarnitine / Sphingolipid |
| tRNA | 4348 | - | A > G | - | Hydroxyshingomyeline C 22:1 / PhosphatidylCholine diacyl C40:6 | SM (OH) C22:1 / PC aa C40:6 | 0.576 | 5.09*10^-06^ | Sphingolipid / Glycerophospholipid |
| 12SrRNA | 856 | rs1603218502 | A > G | - | Shingomyeline C 16:0 / lysoPhosphatidylCholine acyl C24:0 | SM C16:0 / lysoPC a C24:0 | 0.882 | 5.11*10^-06^ | Sphingolipid / Glycerophospholipid |
| tRNA | 12278 | rs1603223646 | T > C | - | PhosphatidylCholine diacyl C40:3 / PhosphatidylCholine diacyl C28:1 | PC aa C40:3 / PC aa C28:1 | 0.955 | 5.13*10^-06^ | Glycerophospholipid / Glycerophospholipid |
| ND2 | 4795 | - | C > T | - | PhosphatidylCholine diacyl C34:1 / PhosphatidylCholine diacyl C38:0 | PC aa C34:1 / PC aa C38:0 | 0.632 | 5.13*10^-06^ | Glycerophospholipid / Glycerophospholipid |
| ND4 | 11493 | - | G > A | - | PhosphatidylCholine acyl-alkyl C42:2 / PhosphatidylCholine acyl-alkyl C38:4 | PC ae C42:2 / PC ae C38:4 | 0.955 | 5.15*10^-06^ | Glycerophospholipid / Glycerophospholipid |
| CYB | 15428 | rs1603225270 | G > A | missense | PhosphatidylCholine diacyl C36:5 / PhosphatidylCholine diacyl C34:4 | PC aa C36:5 / PC aa C34:4 | 0.518 | 5.17*10^-06^ | Glycerophospholipid / Glycerophospholipid |
| ND5 | 12385 | - | C > T | - | PhosphatidylCholine diacyl C42:4 / PhosphatidylCholine acyl-alkyl C42:1 | PC aa C42:4 / PC ae C42:1 | 1.1 | 5.18*10^-06^ | Glycerophospholipid / Glycerophospholipid |
| tRNA | 5659 | - | A > G | - | PhosphatidylCholine acyl-alkyl C30:0 / PhosphatidylCholine diacyl C40:4 | PC ae C30:0 / PC aa C40:4 | 0.538 | 5.19*10^-06^ | Glycerophospholipid / Glycerophospholipid |
| ND4L | 10645 | - | T > C | - | Shingomyeline C 26:0 / PhosphatidylCholine diacyl C34:1 | SM C26:0 / PC aa C34:1 | 0.621 | 5.21*10^-06^ | Sphingolipid / Glycerophospholipid |
| ND4 | 11199 | - | C > T | - | Hydroxyshingomyeline C 16:1 / PhosphatidylCholine acyl-alkyl C34:2 | SM (OH) C16:1 / PC ae C34:2 | 0.769 | 5.23*10^-06^ | Sphingolipid / Glycerophospholipid |
| tRNA | 5615 | - | A > G | - | PhosphatidylCholine acyl-alkyl C36:2 / lysoPhosphatidylCholine acyl C20:3 | PC ae C36:2 / lysoPC a C20:3 | 0.668 | 5.23*10^-06^ | Glycerophospholipid / Glycerophospholipid |
| 12SrRNA | 873 | - | G > A | - | PhosphatidylCholine acyl-alkyl C40:3 / PhosphatidylCholine diacyl C42:4 | PC ae C40:3 / PC aa C42:4 | 1.148 | 5.23*10^-06^ | Glycerophospholipid / Glycerophospholipid |
| ND5 | 13094 | rs1603224029 | T > C | missense | PhosphatidylCholine acyl-alkyl C36:2 / PhosphatidylCholine diacyl C34:2 | PC ae C36:2 / PC aa C34:2 | 1.12 | 5.27*10^-06^ | Glycerophospholipid / Glycerophospholipid |
| ATP6 | 8643 | - | C > T | - | PhosphatidylCholine acyl-alkyl C34:2 / PhosphatidylCholine acyl-alkyl C38:0 | PC ae C34:2 / PC ae C38:0 | 0.49 | 5.37*10^-06^ | Glycerophospholipid / Glycerophospholipid |
| HVR I | 16281 | - | A > G | - | Octadecenoyl-L-carnitine / Tetradecanoyl-L-carnitine | C18:1 / C14 | 1.219 | 5.49*10^-06^ | Acylcarnitine / Acylcarnitine |
| HVR I | 16281 | - | A > G | - | Hexadecenoyl-L-carnitine / Tetradecanoyl-L-carnitine | C16:1 / C14 | 1.46 | 5.50*10^-06^ | Acylcarnitine / Acylcarnitine |
| 16SrRNA | 2582 | rs28441416 | A > G | - | PhosphatidylCholine acyl-alkyl C36:5 / PhosphatidylCholine acyl-alkyl C42:3 | PC ae C36:5 / PC ae C42:3 | 0.708 | 5.51*10^-06^ | Glycerophospholipid / Glycerophospholipid |
| ND4 | 11709 | - | T > C | - | PhosphatidylCholine acyl-alkyl C40:1 / PhosphatidylCholine diacyl C40:5 | PC ae C40:1 / PC aa C40:5 | 0.764 | 5.56*10^-06^ | Glycerophospholipid / Glycerophospholipid |
| 16SrRNA | 2322 | - | C > T | - | PhosphatidylCholine diacyl C40:3 / Shingomyeline C 16:0 | PC aa C40:3 / SM C16:0 | 0.986 | 5.60*10^-06^ | Glycerophospholipid / Sphingolipid |
| ND5 | 12825 | - | T > C | - | lysoPhosphatidylCholine acyl C14:0 / PhosphatidylCholine diacyl C36:6 | lysoPC a C14:0 / PC aa C36:6 | 0.589 | 5.63*10^-06^ | Glycerophospholipid / Glycerophospholipid |
| ND5 | 12488 | - | C > T | - | PhosphatidylCholine acyl-alkyl C32:1 / Hydroxyhexadecadienyl-L-carnitine | PC ae C32:1 / C16:2-OH | 0.659 | 5.64*10^-06^ | Glycerophospholipid / Acylcarnitine |
| ATP6 | 9045 | - | A > G | - | Tetradecadienyl-L-carnitine / Malonyl-L-carnitine , Hydroxybutyryl-L-carnitine | C14:2 / C4-OH (C3-DC) | 0.311 | 5.65*10^-06^ | Acylcarnitine / Acylcarnitine |
| ND5 | 12825 | - | T > C | - | lysoPhosphatidylCholine acyl C14:0 / PhosphatidylCholine diacyl C28:1 | lysoPC a C14:0 / PC aa C28:1 | 0.728 | 5.69*10^-06^ | Glycerophospholipid / Glycerophospholipid |
| ATP6 | 9070 | rs879190502 | T > C | missense | Hexadecanoyl-L-carnitine / Glutaconyl-L-carnitine | C16 / C5:1-DC | 0.738 | 5.71*10^-06^ | Acylcarnitine / Glycerophospholipid |
| ND4 | 11303 | - | A > G | - | Shingomyeline C 18:0 / Tyrosine | SM C18:0 / Tyr | 0.653 | 5.74*10^-06^ | Sphingolipid / Amino Acid |
| ND2 | 5206 | rs1556422963 | C > T | missense | PhosphatidylCholine diacyl C42:2 / PhosphatidylCholine acyl-alkyl C44:3 | PC aa C42:2 / PC ae C44:3 | 1.848 | 5.75*10^-06^ | Glycerophospholipid / Glycerophospholipid |
| CO3 | 9490 | rs1603222345 | C > T | missense | Shingomyeline C 16:1 / PhosphatidylCholine acyl-alkyl C42:0 | SM C16:1 / PC ae C42:0 | 0.851 | 5.76*10^-06^ | Sphingolipid / Glycerophospholipid |
| CYB | 15504 | - | C > T | - | Methylglutaryl-L-carnitine / PhosphatidylCholine acyl-alkyl C30:2 | C5-M-DC / PC ae C30:2 | 0.663 | 5.84*10^-06^ | Acylcarnitine / Glycerophospholipid |
| ND5 | 13937 | - | A > G | - | PhosphatidylCholine acyl-alkyl C34:3 / Tiglyl-L-carnitine | PC ae C34:3 / C5:1 | 0.592 | 5.95*10^-06^ | Glycerophospholipid / Acylcarnitine |
| ND5 | 14141 | - | T > C | - | PhosphatidylCholine acyl-alkyl C42:4 / Hydroxytetradecadienyl-L-carnitine | PC ae C42:4 / C14:2-OH | 0.73 | 5.95*10^-06^ | Glycerophospholipid / Acylcarnitine |
| ND5 | 14141 | - | T > C | - | PhosphatidylCholine acyl-alkyl C42:4 / Hexenoyl-L-carnitine | PC ae C42:4 / C6:1 | 0.739 | 5.97*10^-06^ | Glycerophospholipid / Acylcarnitine |
| ATP6 | 9061 | rs386829061 | C > T | synonymous | PhosphatidylCholine acyl-alkyl C34:1 / Octadecenoyl-L-carnitine | PC ae C34:1 / C18:1 | 0.752 | 5.99*10^-06^ | Glycerophospholipid / Acylcarnitine |
| CO1 | 6207 | - | T > C | - | Shingomyeline C 18:1 / Glutaconyl-L-carnitine | SM C18:1 / C5:1-DC | 0.654 | 6.00*10^-06^ | Sphingolipid / Glycerophospholipid |
| 12SrRNA | 980 | rs397515731 | T > C | - | Shingomyeline C 24:0 / PhosphatidylCholine acyl-alkyl C40:4 | SM C24:0 / PC ae C40:4 | 1.263 | 6.02*10^-06^ | Sphingolipid / Glycerophospholipid |
| CYB | 15794 | rs1603225498 | A > G | missense | Hydroxyshingomyeline C 16:1 / PhosphatidylCholine acyl-alkyl C40:4 | SM (OH) C16:1 / PC ae C40:4 | 0.876 | 6.03*10^-06^ | Sphingolipid / Glycerophospholipid |
| ND4 | 10985 | - | A > G | - | Propionyl-L-carnitine / PhosphatidylCholine acyl-alkyl C38:0 | C3 / PC ae C38:0 | 0.547 | 6.05*10^-06^ | Acylcarnitine / Glycerophospholipid |
| tRNA | 4348 | - | A > G | - | Hydroxyshingomyeline C 22:2 / PhosphatidylCholine acyl-alkyl C42:0 | SM (OH) C22:2 / PC ae C42:0 | 0.788 | 6.12*10^-06^ | Sphingolipid / Glycerophospholipid |
| 16SrRNA | 2486 | rs1603218758 | T > C | - | PhosphatidylCholine acyl-alkyl C34:1 / Glutaconyl-L-carnitine | PC ae C34:1 / C5:1-DC | 0.61 | 6.13*10^-06^ | Glycerophospholipid / Glycerophospholipid |
| ND5 | 14139 | rs878918283 | A > G | synonymous | PhosphatidylCholine diacyl C42:4 / Shingomyeline C 24:1 | PC aa C42:4 / SM C24:1 | 3.144 | 6.15*10^-06^ | Glycerophospholipid / Sphingolipid |
| CO1 | 6994 | - | A > G | - | PhosphatidylCholine acyl-alkyl C30:0 / lysoPhosphatidylCholine acyl C16:1 | PC ae C30:0 / lysoPC a C16:1 | 0.479 | 6.21*10^-06^ | Glycerophospholipid / Glycerophospholipid |
| ND5 | 13160 | - | C > T | - | PhosphatidylCholine acyl-alkyl C32:2 / PhosphatidylCholine acyl-alkyl C40:1 | PC ae C32:2 / PC ae C40:1 | 0.624 | 6.22*10^-06^ | Glycerophospholipid / Glycerophospholipid |
| 12SrRNA | 1445 | - | G > A | - | PhosphatidylCholine diacyl C40:2 / PhosphatidylCholine diacyl C32:0 | PC aa C40:2 / PC aa C32:0 | 1.027 | 6.22*10^-06^ | Glycerophospholipid / Glycerophospholipid |
| ND2 | 4791 | - | A > G | - | Tryptophan / Methionine | Trp / Met | 1.858 | 6.28*10^-06^ | Amino Acid / Amino Acid |
| ND5 | 14141 | - | T > C | - | Hydroxyshingomyeline C 14:1 / Hydroxytetradecadienyl-L-carnitine | SM (OH) C14:1 / C14:2-OH | 0.706 | 6.33*10^-06^ | Sphingolipid / Acylcarnitine |
| 16SrRNA | 2436 | rs1556422630 | G > A | - | Decadienyl-L-carnitine / Ornithine | C10:2 / Orn | 0.713 | 6.36*10^-06^ | Acylcarnitine / Amino Acid |
| ND4 | 11709 | - | T > C | - | PhosphatidylCholine diacyl C36:4 / PhosphatidylCholine diacyl C40:5 | PC aa C36:4 / PC aa C40:5 | 0.955 | 6.37*10^-06^ | Glycerophospholipid / Glycerophospholipid |
| HVR I | 16299 | rs879021682 | A > G | - | PhosphatidylCholine diacyl C24:0 / PhosphatidylCholine diacyl C42:5 | PC aa C24:0 / PC aa C42:5 | 1.086 | 6.41*10^-06^ | Glycerophospholipid / Glycerophospholipid |
| 12SrRNA | 873 | - | G > A | - | PhosphatidylCholine acyl-alkyl C36:3 / PhosphatidylCholine diacyl C42:4 | PC ae C36:3 / PC aa C42:4 | 0.806 | 6.43*10^-06^ | Glycerophospholipid / Glycerophospholipid |
| CO2 | 7589 | - | G > A | - | PhosphatidylCholine acyl-alkyl C40:2 / PhosphatidylCholine acyl-alkyl C36:1 | PC ae C40:2 / PC ae C36:1 | 1.234 | 6.50*10^-06^ | Glycerophospholipid / Glycerophospholipid |
| ND2 | 4795 | - | C > T | - | PhosphatidylCholine diacyl C28:1 / PhosphatidylCholine diacyl C38:0 | PC aa C28:1 / PC aa C38:0 | 0.648 | 6.50*10^-06^ | Glycerophospholipid / Glycerophospholipid |
| CO3 | 9816 | - | C > T | - | PhosphatidylCholine acyl-alkyl C40:1 / PhosphatidylCholine acyl-alkyl C30:2 | PC ae C40:1 / PC ae C30:2 | 0.785 | 6.55*10^-06^ | Glycerophospholipid / Glycerophospholipid |
| tRNA | 15915 | rs1603225588 | G > A | - | lysoPhosphatidylCholine acyl C16:0 / Hydroxyshingomyeline C 24:1 | lysoPC a C16:0 / SM (OH)C24:1 | 0.693 | 6.57*10^-06^ | Glycerophospholipid / Sphingolipid |
| ND1 | 3694 | - | A > G | - | lysoPhosphatidylCholine acyl C17:0 / Arginine | lysoPC a C17:0 / Arg | 0.709 | 6.60*10^-06^ | Glycerophospholipid / Amino Acid |
| 12SrRNA | 1422 | - | G > A | - | PhosphatidylCholine acyl-alkyl C34:0 / lysoPhosphatidylCholine acyl C24:0 | PC ae C34:0 / lysoPC a C24:0 | 0.829 | 6.65*10^-06^ | Glycerophospholipid / Glycerophospholipid |
| CO2 | 7705 | rs1556423330 | T > C | synonymous | PhosphatidylCholine diacyl C32:0 / PhosphatidylCholine diacyl C36:3 | PC aa C32:0 / PC aa C36:3 | 3.175 | 6.67*10^-06^ | Glycerophospholipid / Glycerophospholipid |
| - | 3305 | rs1603218879 | A > G | - | Hydroxyhexadecadienyl-L-carnitine / Decadienyl-L-carnitine | C16:2-OH / C10:2 | 0.88 | 6.68*10^-06^ | Acylcarnitine / Acylcarnitine |
| ND5 | 12488 | - | C > T | - | PhosphatidylCholine acyl-alkyl C34:1 / Hydroxyhexadecadienyl-L-carnitine | PC ae C34:1 / C16:2-OH | 0.677 | 6.71*10^-06^ | Glycerophospholipid / Acylcarnitine |
| 12SrRNA | 1406 | rs111033322 | T > C | - | PhosphatidylCholine acyl-alkyl C36:0 / PhosphatidylCholine diacyl C40:3 | PC ae C36:0 / PC aa C40:3 | 1.839 | 6.73*10^-06^ | Glycerophospholipid / Glycerophospholipid |
| ND4L | 10690 | - | G > A | - | Hydroxyshingomyeline C 14:1 / PhosphatidylCholine acyl-alkyl C42:5 | SM (OH) C14:1 / PC ae C42:5 | 0.796 | 6.74*10^-06^ | Sphingolipid / Glycerophospholipid |
| HVR I | 16299 | rs879021682 | A > G | - | PhosphatidylCholine diacyl C24:0 / PhosphatidylCholine diacyl C38:5 | PC aa C24:0 / PC aa C38:5 | 1.16 | 6.76*10^-06^ | Glycerophospholipid / Glycerophospholipid |
| ND2 | 4795 | - | C > T | - | PhosphatidylCholine diacyl C32:0 / PhosphatidylCholine diacyl C38:0 | PC aa C32:0 / PC aa C38:0 | 0.709 | 6.78*10^-06^ | Glycerophospholipid / Glycerophospholipid |
| CYB | 15865 | rs879154157 | A > G | synonymous | PhosphatidylCholine acyl-alkyl C34:0 / PhosphatidylCholine diacyl C36:0 | PC ae C34:0 / PC aa C36:0 | 0.672 | 6.80*10^-06^ | Glycerophospholipid / Glycerophospholipid |
| ND2 | 4543 | - | A > G | - | Shingomyeline C 24:1 / Shingomyeline C 16:1 | SM C24:1 / SM C16:1 | 1.147 | 6.80*10^-06^ | Sphingolipid / Sphingolipid |
| ATP8 | 8389 | rs386829032 | A > G | synonymous | lysoPhosphatidylCholine acyl C18:1 / PhosphatidylCholine diacyl C34:3 | lysoPC a C18:1 / PC aa C34:3 | 0.649 | 6.85*10^-06^ | Glycerophospholipid / Glycerophospholipid |
| CO2 | 8010 | rs1603221254 | T > C | missense | PhosphatidylCholine acyl-alkyl C36:0 / PhosphatidylCholine diacyl C42:5 | PC ae C36:0 / PC aa C42:5 | 0.684 | 6.89*10^-06^ | Glycerophospholipid / Glycerophospholipid |
| ND4L | 10645 | - | T > C | - | Shingomyeline C 26:0 / PhosphatidylCholine diacyl C40:5 | SM C26:0 / PC aa C40:5 | 0.536 | 6.91*10^-06^ | Sphingolipid / Glycerophospholipid |
| tRNA | 15915 | rs1603225588 | G > A | - | lysoPhosphatidylCholine acyl C18:0 / Hydroxyshingomyeline C 24:1 | lysoPC a C18:0 / SM (OH) C24:1 | 0.676 | 6.92*10^-06^ | Glycerophospholipid / Sphingolipid |
| ND4L | 10645 | - | T > C | - | Shingomyeline C 26:0 / PhosphatidylCholine diacyl C42:6 | SM C26:0 / PC aa C42:6 | 0.613 | 6.94*10^-06^ | Sphingolipid / Glycerophospholipid |
| ND4 | 11027 | - | T > C | - | PhosphatidylCholine acyl-alkyl C40:4 / PhosphatidylCholine acyl-alkyl C34:2 | PC ae C40:4 / PC ae C34:2 | 0.884 | 7.03*10^-06^ | Glycerophospholipid / Glycerophospholipid |
| ND4 | 11823 | - | T > C | - | Ornithine / Threonine | Orn / Thr | 0.907 | 7.05*10^-06^ | Amino Acid / Amino Acid |
| ND2 | 5492 | rs377109345 | T > C | synonymous | Tetradecadienyl-L-carnitine / PhosphatidylCholine diacyl C40:2 | C14:2 / PC aa C40:2 | 0.511 | 7.07*10^-06^ | Acylcarnitine / Glycerophospholipid |
| 16SrRNA | 2548 | - | C > T | - | Hydroxyhexadecenoyl-L-carnitine / Hydroxytetradecadienyl-L-carnitine | C16:1-OH / C14:2-OH | 0.85 | 7.07*10^-06^ | Acylcarnitine / Acylcarnitine |
| 12SrRNA | 1387 | - | A > G | - | PhosphatidylCholine diacyl C36:0 / PhosphatidylCholine acyl-alkyl C40:6 | PC aa C36:0 / PC ae C40:6 | 1.024 | 7.07*10^-06^ | Glycerophospholipid / Glycerophospholipid |
| ND4 | 11513 | - | C > T | - | Hydroxyshingomyeline C 16:1 / Shingomyeline C 24:0 | SM (OH) C16:1 / SM C24:0 | 0.884 | 7.13*10^-06^ | Sphingolipid / Sphingolipid |
| 12SrRNA | 980 | rs397515731 | T > C | - | Shingomyeline C 24:0 / PhosphatidylCholine acyl-alkyl C38:4 | SM C24:0 / PC ae C38:4 | 1.201 | 7.31*10^-06^ | Sphingolipid / Glycerophospholipid |
| ND2 | 4690 | rs1603219557 | T > C | missense | PhosphatidylCholine acyl-alkyl C36:1 / PhosphatidylCholine diacyl C36:3 | PC ae C36:1 / PC aa C36:3 | 0.943 | 7.32*10^-06^ | Glycerophospholipid / Glycerophospholipid |
| HVR I | 16273 | - | G > A | - | Hydroxyshingomyeline C 16:1 / PhosphatidylCholine diacyl C40:6 | SM (OH) C16:1 / PC aa C40:6 | 0.568 | 7.32*10^-06^ | Sphingolipid / Glycerophospholipid |
| CYB | 15454 | rs879015290 | T > C | synonymous | PhosphatidylCholine diacyl C42:4 / Shingomyeline C 24:1 | PC aa C42:4 / SM C24:1 | 3.006 | 7.34*10^-06^ | Glycerophospholipid / Sphingolipid |
| ND4 | 11493 | - | G > A | - | PhosphatidylCholine diacyl C36:5 / PhosphatidylCholine acyl-alkyl C38:4 | PC aa C36:5 / PC ae C38:4 | 0.482 | 7.35*10^-06^ | Glycerophospholipid / Glycerophospholipid |
| ND4 | 11513 | - | C > T | - | Hydroxyshingomyeline C 14:1 / Shingomyeline C 24:0 | SM (OH) C14:1 / SM C24:0 | 0.843 | 7.36*10^-06^ | Sphingolipid / Sphingolipid |
| ND4L | 10690 | - | G > A | - | Hydroxyshingomyeline C 14:1 / PhosphatidylCholine diacyl C42:1 | SM (OH) C14:1 / PC aa C42:1 | 0.693 | 7.48*10^-06^ | Sphingolipid / Glycerophospholipid |
| CO2 | 8091 | - | G > A | - | Hydroxyhexadecadienyl-L-carnitine / Hexadecenoyl-L-carnitine | C16:2-OH / C16:1 | 0.958 | 7.54*10^-06^ | Acylcarnitine / Acylcarnitine |
| tRNA | 4348 | - | A > G | - | PhosphatidylCholine acyl-alkyl C38:2 / PhosphatidylCholine acyl-alkyl C42:0 | PC ae C38:2 / PC ae C42:0 | 0.891 | 7.60*10^-06^ | Glycerophospholipid / Glycerophospholipid |
| - | 5744 | rs1556423026 | G > A | - | lysoPhosphatidylCholine acyl C28:1 / lysoPhosphatidylCholine acyl C28:0 | lysoPC a C28:1 / lysoPC a C28:0 | 1.124 | 7.62*10^-06^ | Glycerophospholipid / Glycerophospholipid |
| ND4 | 11199 | - | C > T | - | lysoPhosphatidylCholine acyl C17:0 / PhosphatidylCholine acyl-alkyl C36:3 | lysoPC a C17:0 / PC ae C36:3 | 0.61 | 7.67*10^-06^ | Glycerophospholipid / Glycerophospholipid |
| ND4 | 11649 | rs1603223370 | T > C | missense | PhosphatidylCholine acyl-alkyl C40:2 / PhosphatidylCholine acyl-alkyl C34:1 | PC ae C40:2 / PC ae C34:1 | 1.182 | 7.68*10^-06^ | Glycerophospholipid / Glycerophospholipid |
| ND4 | 11875 | rs1556424006 | T > C | synonymous | Shingomyeline C 16:1 / PhosphatidylCholine acyl-alkyl C38:5 | SM C16:1 / PC ae C38:5 | 0.984 | 7.69*10^-06^ | Sphingolipid / Glycerophospholipid |
| ATP6 | 9061 | rs386829061 | C > T | synonymous | PhosphatidylCholine acyl-alkyl C34:0 / Hexadecanoyl-L-carnitine | PC ae C34:0 / C16 | 0.755 | 7.75*10^-06^ | Glycerophospholipid / Acylcarnitine |
| 12SrRNA | 873 | - | G > A | - | PhosphatidylCholine acyl-alkyl C36:3 / PhosphatidylCholine diacyl C40:1 | PC ae C36:3 / PC aa C40:1 | 0.856 | 7.77*10^-06^ | Glycerophospholipid / Glycerophospholipid |
| 16SrRNA | 2302 | - | T > C | - | Shingomyeline C 18:1 / DL-Carnitine | SM C18:1 / C0 | 0.716 | 7.77*10^-06^ | Sphingolipid / Acylcarnitine |
| ND4 | 11069 | rs1603223091 | A > G | missense | Shingomyeline C 18:1 / Shingomyeline C 26:0 | SM C18:1 / SM C26:0 | 0.816 | 7.80*10^-06^ | Sphingolipid / Sphingolipid |
| 16SrRNA | 2000 | rs1603218657 | C > T | - | PhosphatidylCholine acyl-alkyl C40:4 / PhosphatidylCholine acyl-alkyl C38:5 | PC ae C40:4 / PC ae C38:5 | 1.218 | 7.81*10^-06^ | Glycerophospholipid / Glycerophospholipid |
| tRNA | 4348 | - | A > G | - | lysoPhosphatidylCholine acyl C28:1 / PhosphatidylCholine diacyl C40:6 | lysoPC a C28:1 / PC aa C40:6 | 0.534 | 7.82*10^-06^ | Glycerophospholipid / Glycerophospholipid |
| ND2 | 4902 | rs1603219664 | A > G | missense | PhosphatidylCholine acyl-alkyl C42:1 / PhosphatidylCholine diacyl C36:4 | PC ae C42:1 / PC aa C36:4 | 0.96 | 7.83*10^-06^ | Glycerophospholipid / Glycerophospholipid |
| ND4L | 10645 | - | T > C | - | Shingomyeline C 26:0 / PhosphatidylCholine acyl-alkyl C38:6 | SM C26:0 / PC ae C38:6 | 0.655 | 7.85*10^-06^ | Sphingolipid / Glycerophospholipid |
| ND2 | 5139 | - | A > G | - | lysoPhosphatidylCholine acyl C28:1 / Octadecadienyl-L-carnitine | lysoPC a C28:1 / C18:2 | 0.524 | 7.91*10^-06^ | Glycerophospholipid / Acylcarnitine |
| CO2 | 8095 | rs1603221283 | A > G | synonymous | PhosphatidylCholine acyl-alkyl C30:2 / Hydroxyshingomyeline C 22:1 | PC ae C30:2 / SM (OH) C22:1 | 1.233 | 7.96*10^-06^ | Glycerophospholipid / Sphingolipid |
| CO2 | 8095 | rs1603221283 | A > G | synonymous | PhosphatidylCholine acyl-alkyl C30:0 / PhosphatidylCholine acyl-alkyl C34:1 | PC ae C30:0 / PC ae C34:1 | 1.723 | 7.96*10^-06^ | Glycerophospholipid / Glycerophospholipid |
| HVR I | 16152 | rs1603225679 | T > C | - | PhosphatidylCholine acyl-alkyl C30:2 / Shingomyeline C 24:1 | PC ae C30:2 / SM C24:1 | 0.671 | 7.96*10^-06^ | Glycerophospholipid / Sphingolipid |
| CYB | 15355 | rs527236181 | G > A | synonymous | Shingomyeline C 24:0 / lysoPhosphatidylCholine acyl C18:0 | SM C24:0 / lysoPC a C18:0 | 1.312 | 7.98*10^-06^ | Sphingolipid / Glycerophospholipid |
| ND4 | 11088 | - | T > C | - | Decanoyl-L-carnitine / Hydroxyhexadecadienyl-L-carnitine | C10 / C16:2-OH | 0.517 | 7.99*10^-06^ | Acylcarnitine / Acylcarnitine |
| CO2 | 8020 | rs879077802 | G > A | synonymous | Hydroxyshingomyeline C 16:1 / lysoPhosphatidylCholine acyl C28:0 | SM (OH) C16:1 / lysoPC a C28:0 | 0.603 | 8.07*10^-06^ | Sphingolipid / Glycerophospholipid |
| ND5 | 13528 | rs55882959 | A > G | missense | PhosphatidylCholine acyl-alkyl C40:2 / PhosphatidylCholine acyl-alkyl C40:3 | PC ae C40:2 / PC ae C40:3 | 1.874 | 8.08*10^-06^ | Glycerophospholipid / Glycerophospholipid |
| HVR II | 115 | rs1603218301 | T > C | - | PhosphatidylCholine diacyl C42:5 / PhosphatidylCholine acyl-alkyl C40:6 | PC aa C42:5 / PC ae C40:6 | 0.896 | 8.11*10^-06^ | Glycerophospholipid / Glycerophospholipid |
| ND4 | 10962 | - | T > C | - | PhosphatidylCholine acyl-alkyl C34:1 / PhosphatidylCholine diacyl C40:1 | PC ae C34:1 / PC aa C40:1 | 0.927 | 8.12*10^-06^ | Glycerophospholipid / Glycerophospholipid |
| ATP6 | 9061 | rs386829061 | C > T | synonymous | PhosphatidylCholine acyl-alkyl C34:1 / Hexadecanoyl-L-carnitine | PC ae C34:1 / C16 | 0.859 | 8.13*10^-06^ | Glycerophospholipid / Acylcarnitine |
| ND4 | 11199 | - | C > T | - | Hydroxyshingomyeline C 16:1 / PhosphatidylCholine acyl-alkyl C36:3 | SM (OH) C16:1 / PC ae C36:3 | 0.734 | 8.20*10^-06^ | Sphingolipid / Glycerophospholipid |
| CO1 | 5972 | rs1603220208 | C > T | synonymous | Hexenoyl-L-carnitine / Fumaryl-L-carnitine , Hexanoyl-L-carnitine | C6:1 / C6 (C4:1-DC) | 1.071 | 8.31*10^-06^ | Acylcarnitine / Acylcarnitine |
| ND2 | 4536 | - | A > G | - | PhosphatidylCholine acyl-alkyl C40:3 / Hydroxyshingomyeline C 22:2 | PC ae C40:3 / SM (OH) C22:2 | 1.08 | 8.33*10^-06^ | Glycerophospholipid / Sphingolipid |
| CO1 | 6207 | - | T > C | - | PhosphatidylCholine acyl-alkyl C38:3 / Glutaconyl-L-carnitine | PC ae C38:3 / C5:1-DC | 0.671 | 8.33*10^-06^ | Glycerophospholipid / Glycerophospholipid |
| CO1 | 6207 | - | T > C | - | Hydroxyshingomyeline C 14:1 / Glutaconyl-L-carnitine | SM (OH) C14:1 / C5:1-DC | 0.624 | 8.36*10^-06^ | Sphingolipid / Glycerophospholipid |
| ND4 | 11172 | rs2853489 | A > G | missense | PhosphatidylCholine acyl-alkyl C42:3 / PhosphatidylCholine diacyl C38:6 | PC ae C42:3 / PC aa C38:6 | 1.605 | 8.40*10^-06^ | Glycerophospholipid / Glycerophospholipid |
| 12SrRNA | 1196 | - | A > G | - | PhosphatidylCholine acyl-alkyl C44:5 / Pimelyl-L-carnitine | PC ae C44:5 / C7-DC | 0.475 | 8.40*10^-06^ | Glycerophospholipid / Acylcarnitine |
| ND1 | 3520 | rs1603218996 | A > G | missense | PhosphatidylCholine diacyl C42:1 / Shingomyeline C 24:0 | PC aa C42:1 / SM C24:0 | 0.648 | 8.44*10^-06^ | Glycerophospholipid / Sphingolipid |
| ND4 | 11513 | - | C > T | - | Hydroxyshingomyeline C 14:1 / Hydroxyshingomyeline C 22:1 | SM (OH) C14:1 / SM (OH) C22:1 | 1.232 | 8.52*10^-06^ | Sphingolipid / Sphingolipid |
| CO1 | 6503 | rs1603220510 | A > G | synonymous | PhosphatidylCholine acyl-alkyl C38:1 / PhosphatidylCholine acyl-alkyl C44:4 | PC ae C38:1 / PC ae C44:4 | 0.558 | 8.52*10^-06^ | Glycerophospholipid / Glycerophospholipid |
| tRNA | 4348 | - | A > G | - | PhosphatidylCholine diacyl C28:1 / PhosphatidylCholine diacyl C40:6 | PC aa C28:1 / PC aa C40:6 | 0.583 | 8.55*10^-06^ | Glycerophospholipid / Glycerophospholipid |
| 12SrRNA | 856 | rs1603218502 | A > G | - | Hydroxyshingomyeline C 22:2 / lysoPhosphatidylCholine acyl C28:1 | SM (OH) C22:2 / lysoPC a C28:1 | 0.847 | 8.59*10^-06^ | Sphingolipid / Glycerophospholipid |
| CYB | 15811 | - | C > T | - | PhosphatidylCholine diacyl C32:3 / PhosphatidylCholine diacyl C38:3 | PC aa C32:3 / PC aa C38:3 | 0.794 | 8.61*10^-06^ | Glycerophospholipid / Glycerophospholipid |
| CO1 | 6856 | rs1603220654 | T > C | missense | PhosphatidylCholine acyl-alkyl C40:3 / PhosphatidylCholine diacyl C36:2 | PC ae C40:3 / PC aa C36:2 | 0.882 | 8.64*10^-06^ | Glycerophospholipid / Glycerophospholipid |
| HVR II | 269 | - | C > T | - | PhosphatidylCholine acyl-alkyl C38:4 / Glycine | PC ae C38:4 / Gly | 0.553 | 8.65*10^-06^ | Glycerophospholipid / Amino Acid |
| ND4L | 10645 | - | T > C | - | Shingomyeline C 26:0 / PhosphatidylCholine acyl-alkyl C36:5 | SM C26:0 / PC ae C36:5 | 0.621 | 8.75*10^-06^ | Sphingolipid / Glycerophospholipid |
| ATP8 | 8389 | rs386829032 | A > G | synonymous | lysoPhosphatidylCholine acyl C18:1 / PhosphatidylCholine acyl-alkyl C36:5 | lysoPC a C18:1 / PC ae C36:5 | 0.611 | 8.79*10^-06^ | Glycerophospholipid / Glycerophospholipid |
| CO3 | 9564 | rs1603222373 | G > A | missense | PhosphatidylCholine diacyl C34:2 / Tetradecenoyl-L-carnitine | PC aa C34:2 / C14:1 | 0.876 | 8.80*10^-06^ | Glycerophospholipid / Acylcarnitine |
| ND4 | 11307 | - | C > T | - | PhosphatidylCholine diacyl C36:1 / Shingomyeline C 16:1 | PC aa C36:1 / SM C16:1 | 0.93 | 8.86*10^-06^ | Glycerophospholipid / Sphingolipid |
| ND5 | 12909 | rs1603223967 | A > G | synonymous | PhosphatidylCholine diacyl C38:5 / PhosphatidylCholine acyl-alkyl C34:3 | PC aa C38:5 / PC ae C34:3 | 0.604 | 8.94*10^-06^ | Glycerophospholipid / Glycerophospholipid |
| ND4 | 11191 | rs1556423907 | C > T | synonymous | PhosphatidylCholine diacyl C34:1 / PhosphatidylCholine diacyl C38:3 | PC aa C34:1 / PC aa C38:3 | 1.423 | 9.02*10^-06^ | Glycerophospholipid / Glycerophospholipid |
| ND5 | 14005 | - | T > C | - | PhosphatidylCholine acyl-alkyl C44:5 / PhosphatidylCholine acyl-alkyl C32:2 | PC ae C44:5 / PC ae C32:2 | 0.711 | 9.07*10^-06^ | Glycerophospholipid / Glycerophospholipid |
| CO1 | 6856 | rs1603220654 | T > C | missense | PhosphatidylCholine acyl-alkyl C30:2 / PhosphatidylCholine diacyl C36:2 | PC ae C30:2 / PC aa C36:2 | 0.757 | 9.09*10^-06^ | Glycerophospholipid / Glycerophospholipid |
| - | 426 | - | A > G | - | PhosphatidylCholine diacyl C38:0 / Tetradecenoyl-L-carnitine | PC aa C38:0 / C14:1 | 0.727 | 9.10*10^-06^ | Glycerophospholipid / Acylcarnitine |
| CO1 | 6474 | - | A > G | - | xLeucine / Glutamine | xLeu / Gln | 1.126 | 9.13*10^-06^ | Amino Acid / Amino Acid |
| ND4 | 11493 | - | G > A | - | PhosphatidylCholine diacyl C36:6 / PhosphatidylCholine acyl-alkyl C38:4 | PC aa C36:6 / PC ae C38:4 | 0.534 | 9.13*10^-06^ | Glycerophospholipid / Glycerophospholipid |
| tRNA | 5615 | - | A > G | - | PhosphatidylCholine acyl-alkyl C34:3 / lysoPhosphatidylCholine acyl C20:3 | PC ae C34:3 / lysoPC a C20:3 | 0.6 | 9.16*10^-06^ | Glycerophospholipid / Glycerophospholipid |
| 12SrRNA | 856 | rs1603218502 | A > G | - | PhosphatidylCholine acyl-alkyl C40:2 / lysoPhosphatidylCholine acyl C28:1 | PC ae C40:2 / lysoPC a C28:1 | 0.833 | 9.27*10^-06^ | Glycerophospholipid / Glycerophospholipid |
| 16SrRNA | 1957 | - | A > G | - | PhosphatidylCholine acyl-alkyl C44:6 / PhosphatidylCholine acyl-alkyl C38:5 | PC ae C44:6 / PC ae C38:5 | 0.799 | 9.27*10^-06^ | Glycerophospholipid / Glycerophospholipid |
| HVR I | 16116 | - | A > G | - | DL-Carnitine / PhosphatidylCholine diacyl C36:3 | C0 / PC aa C36:3 | 0.799 | 9.31*10^-06^ | Acylcarnitine / Glycerophospholipid |
| 12SrRNA | 1422 | - | G > A | - | PhosphatidylCholine acyl-alkyl C30:0 / lysoPhosphatidylCholine acyl C28:1 | PC ae C30:0 / lysoPC a C28:1 | 0.997 | 9.32*10^-06^ | Glycerophospholipid / Glycerophospholipid |
| tRNA | 4348 | - | A > G | - | Hydroxyshingomyeline C 22:2 / PhosphatidylCholine diacyl C40:6 | SM (OH) C22:2 / PC aa C40:6 | 0.548 | 9.48*10^-06^ | Sphingolipid / Glycerophospholipid |
| ATP8 | 8477 | rs1603221517 | T > C | missense | PhosphatidylCholine diacyl C40:6 / PhosphatidylCholine diacyl C40:2 | PC aa C40:6 / PC aa C40:2 | 0.458 | 9.62*10^-06^ | Glycerophospholipid / Glycerophospholipid |
| - | 426 | - | A > G | - | PhosphatidylCholine diacyl C38:0 / Hydroxytetradecadienyl-L-carnitine | PC aa C38:0 / C14:2-OH | 0.684 | 9.66*10^-06^ | Glycerophospholipid / Acylcarnitine |
| HVR II | 125 | rs144402189 | T > C | - | PhosphatidylCholine acyl-alkyl C36:0 / PhosphatidylCholine acyl-alkyl C34:3 | PC ae C36:0 / PC ae C34:3 | 1.002 | 9.73*10^-06^ | Glycerophospholipid / Glycerophospholipid |
| CO3 | 9441 | - | C > T | - | PhosphatidylCholine acyl-alkyl C42:4 / PhosphatidylCholine diacyl C30:0 | PC ae C42:4 / PC aa C30:0 | 0.539 | 9.79*10^-06^ | Glycerophospholipid / Glycerophospholipid |
| tRNA | 7503 | - | C > T | - | PhosphatidylCholine acyl-alkyl C42:2 / lysoPhosphatidylCholine acyl C20:4 | PC ae C42:2 / lysoPC a C20:4 | 0.498 | 9.80*10^-06^ | Glycerophospholipid / Glycerophospholipid |
| 12SrRNA | 715 | - | G > A | - | Valeryl-L-carnitine / Decenoyl-L-carnitine | C5 / C10:1 | 0.626 | 9.82*10^-06^ | Acylcarnitine / Acylcarnitine |
| HVR I | 16092 | rs1556424740 | T > C | - | PhosphatidylCholine acyl-alkyl C44:6 / PhosphatidylCholine diacyl C42:1 | PC ae C44:6 / PC aa C42:1 | 4.494 | 9.83*10^-06^ | Glycerophospholipid / Glycerophospholipid |
| 12SrRNA | 1028 | - | G > A | - | PhosphatidylCholine acyl-alkyl C38:1 / PhosphatidylCholine acyl-alkyl C34:3 | PC ae C38:1 / PC ae C34:3 | 0.498 | 9.85*10^-06^ | Glycerophospholipid / Glycerophospholipid |
| ND1 | 4175 | - | G > A | - | Hydroxyshingomyeline C 16:1 / Octadecanoyl-L-carnitine | SM (OH) C16:1 / C18 | 0.619 | 9.88*10^-06^ | Sphingolipid / Acylcarnitine |
| ND5 | 14141 | - | T > C | - | PhosphatidylCholine acyl-alkyl C36:2 / Hexenoyl-L-carnitine | PC ae C36:2 / C6:1 | 0.738 | 9.97*10^-06^ | Glycerophospholipid / Acylcarnitine |
| tRNA | 5564 | - | C > T | - | PhosphatidylCholine diacyl C30:0 / Shingomyeline C 26:1 | PC aa C30:0 / SM C26:1 | 0.567 | 1.00*10^-05^ | Glycerophospholipid / Sphingolipid |
| ND5 | 13485 | rs28359176 | A > G | synonymous | lysoPhosphatidylCholine acyl C16:0 / PhosphatidylCholine acyl-alkyl C42:3 | lysoPC a C16:0 / PC ae C42:3 | 0.8 | 1.01*10^-05^ | Glycerophospholipid / Glycerophospholipid |
| ND1 | 3310 | rs1603218889 | C > T | missense | PhosphatidylCholine acyl-alkyl C36:4 / PhosphatidylCholine acyl-alkyl C38:1 | PC ae C36:4 / PC ae C38:1 | 0.57 | 1.01*10^-05^ | Glycerophospholipid / Glycerophospholipid |
| HVR I | 16299 | rs879021682 | A > G | - | lysoPhosphatidylCholine acyl C28:0 / PhosphatidylCholine diacyl C42:6 | lysoPC a C28:0 / PC aa C42:6 | 1.451 | 1.01*10^-05^ | Glycerophospholipid / Glycerophospholipid |
| ND5 | 13030 | - | T > C | - | PhosphatidylCholine acyl-alkyl C44:3 / PhosphatidylCholine diacyl C42:4 | PC ae C44:3 / PC aa C42:4 | 0.988 | 1.01*10^-05^ | Glycerophospholipid / Glycerophospholipid |
| ND4 | 11088 | - | T > C | - | Dodecanoyl-L-carnitine / Hydroxyhexadecadienyl-L-carnitine | C12 / C16:2-OH | 0.589 | 1.01*10^-05^ | Acylcarnitine / Acylcarnitine |
| CO2 | 7976 | rs377368526 | G > A | missense | PhosphatidylCholine acyl-alkyl C36:1 / PhosphatidylCholine acyl-alkyl C38:0 | PC ae C36:1 / PC ae C38:0 | 0.925 | 1.02*10^-05^ | Glycerophospholipid / Glycerophospholipid |
| 12SrRNA | 856 | rs1603218502 | A > G | - | Shingomyeline C 16:0 / lysoPhosphatidylCholine acyl C28:1 | SM C16:0 / lysoPC a C28:1 | 0.794 | 1.02*10^-05^ | Sphingolipid / Glycerophospholipid |
| CO2 | 8014 | rs879223416 | A > G | synonymous | PhosphatidylCholine diacyl C36:0 / Shingomyeline C 26:1 | PC aa C36:0 / SM C26:1 | 0.696 | 1.03*10^-05^ | Glycerophospholipid / Sphingolipid |
| 16SrRNA | 2763 | - | T > C | - | PhosphatidylCholine acyl-alkyl C34:0 / PhosphatidylCholine diacyl C32:0 | PC ae C34:0 / PC aa C32:0 | 0.914 | 1.03*10^-05^ | Glycerophospholipid / Glycerophospholipid |
| ND4 | 11199 | - | C > T | - | PhosphatidylCholine acyl-alkyl C36:1 / PhosphatidylCholine acyl-alkyl C36:3 | PC ae C36:1 / PC ae C36:3 | 0.833 | 1.04*10^-05^ | Glycerophospholipid / Glycerophospholipid |
| ND4 | 11493 | - | G > A | - | PhosphatidylCholine acyl-alkyl C34:0 / PhosphatidylCholine acyl-alkyl C38:4 | PC ae C34:0 / PC ae C38:4 | 0.935 | 1.05*10^-05^ | Glycerophospholipid / Glycerophospholipid |
| ND5 | 14141 | - | T > C | - | lysoPhosphatidylCholine acyl C17:0 / Hexenoyl-L-carnitine | lysoPC a C17:0 / C6:1 | 0.637 | 1.05*10^-05^ | Glycerophospholipid / Acylcarnitine |
| ND4 | 10962 | - | T > C | - | PhosphatidylCholine acyl-alkyl C34:1 / Hydroxyhexadecadienyl-L-carnitine | PC ae C34:1 / C16:2-OH | 0.752 | 1.05*10^-05^ | Glycerophospholipid / Acylcarnitine |
| ND1 | 3694 | - | A > G | - | PhosphatidylCholine acyl-alkyl C38:4 / Shingomyeline C 16:0 | PC ae C38:4 / SM C16:0 | 1.171 | 1.05*10^-05^ | Glycerophospholipid / Sphingolipid |
| ATP6 | 9061 | rs386829061 | C > T | synonymous | PhosphatidylCholine diacyl C30:0 / Hexadecanoyl-L-carnitine | PC aa C30:0 / C16 | 0.635 | 1.05*10^-05^ | Glycerophospholipid / Acylcarnitine |
| ND5 | 14141 | - | T > C | - | PhosphatidylCholine diacyl C42:4 / Hexenoyl-L-carnitine | PC aa C42:4 / C6:1 | 0.809 | 1.05*10^-05^ | Glycerophospholipid / Acylcarnitine |
| ND4 | 11493 | - | G > A | - | PhosphatidylCholine diacyl C40:3 / PhosphatidylCholine acyl-alkyl C38:4 | PC aa C40:3 / PC ae C38:4 | 0.935 | 1.07*10^-05^ | Glycerophospholipid / Glycerophospholipid |
| ND5 | 13337 | - | T > C | - | Hydroxyshingomyeline C 16:1 / Hexadecanoyl-L-carnitine | SM (OH) C16:1 / C16 | 0.679 | 1.07*10^-05^ | Sphingolipid / Acylcarnitine |
| CYB | 15141 | rs1603225108 | T > C | missense | lysoPhosphatidylCholine acyl C17:0 / PhosphatidylCholine acyl-alkyl C30:2 | lysoPC a C17:0 / PC ae C30:2 | 0.514 | 1.07*10^-05^ | Glycerophospholipid / Glycerophospholipid |
| ND4L | 10645 | - | T > C | - | Shingomyeline C 26:0 / PhosphatidylCholine diacyl C36:6 | SM C26:0 / PC aa C36:6 | 0.452 | 1.08*10^-05^ | Sphingolipid / Glycerophospholipid |
| 16SrRNA | 2582 | rs28441416 | A > G | - | PhosphatidylCholine acyl-alkyl C36:5 / PhosphatidylCholine acyl-alkyl C38:0 | PC ae C36:5 / PC ae C38:0 | 0.64 | 1.08*10^-05^ | Glycerophospholipid / Glycerophospholipid |
| CYB | 15454 | rs879015290 | T > C | synonymous | PhosphatidylCholine diacyl C42:4 / Shingomyeline C 16:0 | PC aa C42:4 / SM C16:0 | 3.286 | 1.08*10^-05^ | Glycerophospholipid / Sphingolipid |
| ND1 | 3694 | - | A > G | - | PhosphatidylCholine acyl-alkyl C38:4 / Shingomyeline C 24:1 | PC ae C38:4 / SM C24:1 | 0.931 | 1.09*10^-05^ | Glycerophospholipid / Sphingolipid |
| HVR I | 16068 | - | T > C | - | PhosphatidylCholine acyl-alkyl C30:2 / PhosphatidylCholine acyl-alkyl C36:2 | PC ae C30:2 / PC ae C36:2 | 1.275 | 1.09*10^-05^ | Glycerophospholipid / Glycerophospholipid |
| tRNA | 5799 | - | A > G | - | Shingomyeline C 20:2 / Hydroxyshingomyeline C 22:2 | SM C20:2 / SM (OH) C22:2 | 0.753 | 1.10*10^-05^ | Sphingolipid / Sphingolipid |
| 16SrRNA | 2486 | rs1603218758 | T > C | - | lysoPhosphatidylCholine acyl C16:1 / Glutaconyl-L-carnitine | lysoPC a C16:1 / C5:1-DC | 0.47 | 1.10*10^-05^ | Glycerophospholipid / Glycerophospholipid |
| CO3 | 9986 | rs879123897 | G > A | synonymous | PhosphatidylCholine diacyl C36:1 / Shingomyeline C 24:0 | PC aa C36:1 / SM C24:0 | 1.371 | 1.11*10^-05^ | Glycerophospholipid / Sphingolipid |
| ND5 | 13485 | rs28359176 | A > G | synonymous | lysoPhosphatidylCholine acyl C18:0 / PhosphatidylCholine acyl-alkyl C42:3 | lysoPC a C18:0 / PC ae C42:3 | 0.741 | 1.11*10^-05^ | Glycerophospholipid / Glycerophospholipid |
| ND4 | 11902 | rs1603223459 | G > A | synonymous | PhosphatidylCholine acyl-alkyl C36:2 / PhosphatidylCholine acyl-alkyl C38:6 | PC ae C36:2 / PC ae C38:6 | 0.758 | 1.12*10^-05^ | Glycerophospholipid / Glycerophospholipid |
| - | 5744 | rs1556423026 | G > A | - | Hydroxyshingomyeline C 16:1 / lysoPhosphatidylCholine acyl C28:0 | SM (OH) C16:1 / lysoPC a C28:0 | 0.585 | 1.13*10^-05^ | Sphingolipid / Glycerophospholipid |
| tRNA | 5709 | - | T > C | - | Hydroxyshingomyeline C 16:1 / PhosphatidylCholine diacyl C32:3 | SM (OH) C16:1 / PC aa C32:3 | 0.846 | 1.13*10^-05^ | Sphingolipid / Glycerophospholipid |
| ND6 | 14623 | - | C > T | - | Shingomyeline C 24:1 / PhosphatidylCholine acyl-alkyl C36:5 | SM C24:1 / PC ae C36:5 | 1.034 | 1.14*10^-05^ | Sphingolipid / Glycerophospholipid |
| ND5 | 14005 | - | T > C | - | PhosphatidylCholine acyl-alkyl C44:5 / PhosphatidylCholine diacyl C42:4 | PC ae C44:5 / PC aa C42:4 | 0.69 | 1.15*10^-05^ | Glycerophospholipid / Glycerophospholipid |
| tRNA | 10031 | rs200048690 | T > C | - | Octanoyl-L-carnitine / Hexadecanoyl-L-carnitine | C8 / C16 | 0.952 | 1.15*10^-05^ | Acylcarnitine / Acylcarnitine |
| ND5 | 13485 | rs28359176 | A > G | synonymous | lysoPhosphatidylCholine acyl C18:0 / PhosphatidylCholine diacyl C38:0 | lysoPC a C18:0 / PC aa C38:0 | 0.653 | 1.15*10^-05^ | Glycerophospholipid / Glycerophospholipid |
| HVR I | 16082 | - | C > T | - | PhosphatidylCholine acyl-alkyl C38:3 / PhosphatidylCholine acyl-alkyl C40:2 | PC ae C38:3 / PC ae C40:2 | 1.014 | 1.15*10^-05^ | Glycerophospholipid / Glycerophospholipid |
| ND4 | 11506 | rs878894616 | T > C | synonymous | Hydroxyshingomyeline C 22:1 / PhosphatidylCholine diacyl C36:1 | SM (OH) C22:1 / PC aa C36:1 | 0.744 | 1.16*10^-05^ | Sphingolipid / Glycerophospholipid |
| CYB | 15865 | rs879154157 | A > G | synonymous | PhosphatidylCholine diacyl C36:1 / PhosphatidylCholine diacyl C36:0 | PC aa C36:1 / PC aa C36:0 | 0.598 | 1.18*10^-05^ | Glycerophospholipid / Glycerophospholipid |
| 16SrRNA | 2363 | - | A > G | - | PhosphatidylCholine diacyl C30:0 / PhosphatidylCholine diacyl C32:1 | PC aa C30:0 / PC aa C32:1 | 0.663 | 1.18*10^-05^ | Glycerophospholipid / Glycerophospholipid |
| CO2 | 8095 | rs1603221283 | A > G | synonymous | PhosphatidylCholine acyl-alkyl C30:2 / PhosphatidylCholine acyl-alkyl C38:4 | PC ae C30:2 / PC ae C38:4 | 1.147 | 1.18*10^-05^ | Glycerophospholipid / Glycerophospholipid |
| CO3 | 9389 | rs28462217 | A > G | synonymous | PhosphatidylCholine diacyl C28:1 / PhosphatidylCholine diacyl C36:3 | PC aa C28:1 / PC aa C36:3 | 1.396 | 1.19*10^-05^ | Glycerophospholipid / Glycerophospholipid |
| ND6 | 14623 | - | C > T | - | Shingomyeline C 26:1 / PhosphatidylCholine acyl-alkyl C34:3 | SM C26:1 / PC ae C34:3 | 0.798 | 1.19*10^-05^ | Sphingolipid / Glycerophospholipid |
| CO2 | 7589 | - | G > A | - | PhosphatidylCholine acyl-alkyl C40:2 / PhosphatidylCholine diacyl C36:2 | PC ae C40:2 / PC aa C36:2 | 0.843 | 1.19*10^-05^ | Glycerophospholipid / Glycerophospholipid |
| ND5 | 13373 | - | T > C | - | PhosphatidylCholine acyl-alkyl C40:3 / Hydroxyshingomyeline C 22:1 | PC ae C40:3 / SM (OH) C22:1 | 0.902 | 1.19*10^-05^ | Glycerophospholipid / Sphingolipid |
| 12SrRNA | 1406 | rs111033322 | T > C | - | PhosphatidylCholine acyl-alkyl C36:0 / PhosphatidylCholine diacyl C40:1 | PC ae C36:0 / PC aa C40:1 | 1.759 | 1.20*10^-05^ | Glycerophospholipid / Glycerophospholipid |
| tRNA | 4418 | rs1556422856 | T > C | - | Shingomyeline C 24:1 / lysoPhosphatidylCholine acyl C18:0 | SM C24:1 / lysoPC a C18:0 | 1.084 | 1.20*10^-05^ | Sphingolipid / Glycerophospholipid |
| ND1 | 3310 | rs1603218889 | C > T | missense | PhosphatidylCholine acyl-alkyl C38:5 / PhosphatidylCholine acyl-alkyl C38:1 | PC ae C38:5 / PC ae C38:1 | 0.607 | 1.20*10^-05^ | Glycerophospholipid / Glycerophospholipid |
| ND2 | 4586 | rs372070966 | T > C | synonymous | PhosphatidylCholine acyl-alkyl C40:1 / PhosphatidylCholine diacyl C40:6 | PC ae C40:1 / PC aa C40:6 | 0.54 | 1.20*10^-05^ | Glycerophospholipid / Glycerophospholipid |
| CO2 | 8141 | rs1603221309 | G > A | missense | Shingomyeline C 16:0 / PhosphatidylCholine acyl-alkyl C36:0 | SM C16:0 / PC ae C36:0 | 0.943 | 1.20*10^-05^ | Sphingolipid / Glycerophospholipid |
| CO1 | 6331 | - | T > C | - | PhosphatidylCholine acyl-alkyl C36:1 / lysoPhosphatidylCholine acyl C24:0 | PC ae C36:1 / lysoPC a C24:0 | 0.86 | 1.21*10^-05^ | Glycerophospholipid / Glycerophospholipid |
| CO2 | 8095 | rs1603221283 | A > G | synonymous | lysoPhosphatidylCholine acyl C24:0 / PhosphatidylCholine diacyl C36:4 | lysoPC a C24:0 / PC aa C36:4 | 1.019 | 1.21*10^-05^ | Glycerophospholipid / Glycerophospholipid |
| tRNA | 12170 | rs1556424072 | G > A | - | PhosphatidylCholine acyl-alkyl C38:3 / PhosphatidylCholine diacyl C36:2 | PC ae C38:3 / PC aa C36:2 | 0.973 | 1.21*10^-05^ | Glycerophospholipid / Glycerophospholipid |
| ATP6 | 8736 | rs879110334 | T > C | synonymous | PhosphatidylCholine diacyl C32:3 / PhosphatidylCholine diacyl C42:6 | PC aa C32:3 / PC aa C42:6 | 0.872 | 1.23*10^-05^ | Glycerophospholipid / Glycerophospholipid |
| - | 426 | - | A > G | - | PhosphatidylCholine acyl-alkyl C38:6 / Tetradecenoyl-L-carnitine | PC ae C38:6 / C14:1 | 0.755 | 1.23*10^-05^ | Glycerophospholipid / Acylcarnitine |
| ATP6 | 9061 | rs386829061 | C > T | synonymous | PhosphatidylCholine acyl-alkyl C36:1 / Octadecanoyl-L-carnitine | PC ae C36:1 / C18 | 0.739 | 1.24*10^-05^ | Glycerophospholipid / Acylcarnitine |
| ND2 | 5258 | rs1603219853 | A > G | synonymous | PhosphatidylCholine diacyl C34:1 / Shingomyeline C 24:0 | PC aa C34:1 / SM C24:0 | 1.355 | 1.25*10^-05^ | Glycerophospholipid / Sphingolipid |
| - | 5744 | rs1556423026 | G > A | - | Hydroxyshingomyeline C 24:1 / lysoPhosphatidylCholine acyl C28:0 | SM (OH) C24:1 / lysoPC a C28:0 | 0.608 | 1.25*10^-05^ | Sphingolipid / Glycerophospholipid |
| ND5 | 13002 | rs878926912 | C > T | synonymous | PhosphatidylCholine acyl-alkyl C30:0 / PhosphatidylCholine acyl-alkyl C42:1 | PC ae C30:0 / PC ae C42:1 | 0.565 | 1.25*10^-05^ | Glycerophospholipid / Glycerophospholipid |

**Legend:** Genomic position in base pairs (bp), alleles, rs_number, and point mutation are based on the NCBI dbSNP GRCh38 human genome assembly (rCRS, GeneBank ID NC_012920.1). Alleles are given in terms of major > minor allele. Nominal p-values are provided for each Beta. mtSNP: mitochondrial single nucleotide polymorphism; MT-Gene: mitochondrial gene.
